# Modulation of the cell wall protein Ecm33p in yeast *Saccharomyces cerevisiae* improves the production of small metabolites

**DOI:** 10.1093/femsyr/foac037

**Published:** 2022-08-03

**Authors:** Verónica Ramos-Viana, Iben Møller-Hansen, Paul Kempen, Irina Borodina

**Affiliations:** The Novo Nordisk Foundation Center for Biosustainability, Technical University of Denmark, Kemitorvet 220, DK-2800 Kgs. Lyngby, Denmark; The Novo Nordisk Foundation Center for Biosustainability, Technical University of Denmark, Kemitorvet 220, DK-2800 Kgs. Lyngby, Denmark; Department of Health Technology, Section for Biotherapeutic Engineering and Drug Targeting, Technical University of Denmark, Ørsteds Plads 345C, DK-2800 Kgs. Lyngby, Denmark; National Center for Nano Fabrication and Characterization, Technical University of Denmark, Ørsteds Plads 347, DK-2800 Kgs. Lyngby, Denmark; The Novo Nordisk Foundation Center for Biosustainability, Technical University of Denmark, Kemitorvet 220, DK-2800 Kgs. Lyngby, Denmark

**Keywords:** cell wall, yeast cell factory, cell wall integrity signaling pathway, *ECM33*, calcofluor white

## Abstract

The cell wall is a dynamic organelle that determines the shape and provides the cell with mechanical strength. This study investigated whether modulation of cell wall composition can influence the production or secretion of small metabolites by yeast cell factories. We deleted and upregulated several cell wall-related genes *KRE2*, *CWP1*, *CWP2*, *ECM33*, *PUN1*, and *LAS21* in yeast *Saccharomyces cerevisiae* engineered for *p*-coumaric acid or β-carotene production. Deletions of *las21∆* and *ecm33∆* impaired the yeast growth on medium with cell wall stressors, calcofluor white, and caffeine. Both overexpression and deletion of *ECM33* significantly improved the specific yield of *p*-coumaric acid and β-carotene. We observed no change in secretion in any cell wall-altered mutants, suggesting the cell wall is not a limiting factor for small molecule secretion at the current production levels. We evaluated the cell wall morphology of the *ECM33* mutant strains using transmission electron microscopy. The *ecm33∆* mutants had an increased chitin deposition and a less structured cell wall, while the opposite was observed in *ECM33*-overexpressing strains. Our results point at the cell wall-related gene *ECM33* as a potential target for improving production in engineered yeast cell factories.

## Teaser

Altering the cell wall improves the production of two small molecules, Ï-coumaric acid or Î²-carotene, in yeast cell factories.

**List of abbreviations**
CWI: cell wall integrity
TEM: transmission electron microscopy
CS: chitin synthase
CFW: calcofluor white
FIT: Feed-In-Time medium
HPLC: high-performance liquid chromatography

## Introduction

The yeast *Saccharomyces cerevisiae* is an attractive cell factory due to its history of safe use and genetic manipulation ease. Cell factories have been engineered to produce bulk and high-value chemicals and biofuels (Li and Borodina 2015, Yuan and Alper 2019). When optimizing cell factories, the typical engineering targets are metabolic and sometimes regulatory genes, while the genes related to structural cell components, such as cell wall, are seldom considered. Dahlin et al. (2019) showed that *S. cerevisiae* engineered to produce fatty alcohols overexpressed cell wall stress-related genes. Likewise, exposure to high ethanol concentrations during fermentations also induces the cell wall integrity (CWI) signaling pathway (Udom et al. 2019).

The yeast cell wall is a strong, yet elastic structure protecting the cells from osmotic shock and mechanical stress and maintaining the cell shape essential for cell division. Furthermore, the glycoproteins on the cell surface participate in cell–cell interactions during mating and flocculation. It also limits the entry of macromolecules, such as enzymes that could degrade the plasma membrane (Levin 2011). The inner layer of the cell wall is composed of cross-linked β-1,3- and β-1,6-linked glucans and chitin and provides mechanical strength. However, chitin itself is a minor constituent of the cell wall, with only 1%–2% of the dry weight of the cell wall being chitin. The outer layer of the cell wall is made primarily of mannoproteins (cell wall proteins, CWP), covalently linked to glucans (Lipke and Ovalle 1998). Mannoproteins account for 30%–50% of the cell wall dry weight (Kapteyn et al. 1999a) and have a role in adhesion, cell wall biogenesis, and strengthening or limiting the permeability of the cell wall to macromolecules (Zlotnik et al. 1984, De Nobel et al. 1989, Kapteyn et al. 1999b, Sundstrom 2002). The cell wall is remodeled during growth, cell division, and environmental stress, all processes orchestrated by the CWI signaling pathway (Schiavone et al. 2014, Sanz and Arroyo 2017).

Only a handful of publications involve the perturbation of the cell wall in metabolic engineering, with the focus being on protein secretion. Bartkevičiūtė and Sasnauskas (2004) and Tang et al. (2016) disrupted the mannosyl transferases Och1p, Mnn1p, Mnn9p, and Mnn10p and achieved an improved secretion of heterologous proteins. Likewise, overexpression of *CWP2* also resulted in higher secretion of proteins (Wentz and Shusta 2007). Valachovic et al. (2016) deleted the CWP-encoding gene *ECM33* in a squalene-producing *S. cerevisiae* strain. Surprisingly, the deletion restored membrane rigidity caused by the lipophilic squalene and increased squalene production by 12% (Son et al. 2020). Ecm33p is a known activator of the CWI pathway, and its deletion starts a compensatory mechanism by increasing chitin deposition (Pardo et al. 2004, Arias et al. 2011). The chitin synthase CSIII was subsequently found to be the enzyme responsible for increasing cell wall chitin levels (Valdivieso et al. 2000, de Groot et al. 2001, Lagorce et al. 2003). Likewise, the disruption of *CWP2*, a gene encoding one of the most abundant CWP, was also shown to improve the secretion of heterologous cellulase (Li et al. 2020).

As mentioned previously, overexpression of *CWP2* also resulted in higher secretion of proteins (Wentz and Shusta 2007). The improved protein secretion in the *CWP2* overexpression mutant suggests that overactivation of the CWI pathway makes the cell wall more permeable to protein secretion. In agreement with these findings, the over-activation of the CWI pathway was shown to make cells more susceptible to stressors like acetic acid (Rego et al. 2014).

The conflicting results from the previous studies overexpressing and deleting genes relating to the cell wall suggest complex mechanisms by which the cell wall structure may influence the production of small metabolites in microbial cell factories. In this study, we sought to elucidate further the connection between the cell wall structure, cell wall stress, and the production of small compounds. To modulate the permeability and rigidity of the cell wall, we deleted and overexpressed six cell wall-related genes (*LAS21*, *KRE2*, *ECM33*, *PUN1*, *CWP1*, and *CWP2*). We did so in two strains producing *p*-coumaric acid or β-carotene to investigate the effect of cell wall remodeling on the production of small molecules. We also characterized their response to cell wall stressors and used fluorescence and TEM images to analyze the effect on the cell wall morphology.

## Materials and methods

### Strain construction and cultivation

The background strains in this study were genetically modified previously from the parent strain *S. cerevisiae* CEN.PK113-7D. These include a *p*-coumaric acid producer strain ST10283 *MATa URA3 HIS3 LEU2 TRP1 MAL2-8c SUC2 ΔGPP1 ΔPDC5 ΔARO10 X-4::Aro4^K229L^-Aro7^G141S^ XI-3::ARO1-ARO2 XII-5::ARO3-PHA2 XI-1::FjTAL-EcAroL* (in-house strain) and β-carotene producer strain ST8939 with *MATa URA3 HIS3 LEU2 TRP1 MAL2-8c SUC2 X-4::XdCrtI-XdCrtYB XII-5::XdCrtE XI-3::XdCrtI-tHMG1 *+ pCfB2312 (2 μm cas9 KanMX; Milne et al. 2020). All *S. cerevisiae* strains constructed in this study are derived from ST10283 and ST8939.

Routine growth of *S. cerevisiae* was carried out at 30°C in yeast peptone dextrose (YPD) medium, containing 10 g/l yeast extract, 20 g/l peptone, and 20 g/l D-glucose. In spot assays, yeast strains were grown in YED medium containing 10 g/l yeast extract and 20 g/l D-glucose. A total of 20 g/l agar was added if solid cultivations were required.

The complete list of strains, plasmids, biobricks, and primers used in this work are available in Tables S1–S4 (Supporting Information). Plasmids required for genome engineering were constructed using the set of vectors from EasyClone-MarkerFree as a backbone (Jessop-Fabre et al. 2016). Integrative vector backbones were linearized by digestion, while guide-RNA (gRNA) vector backbones were PCR-amplified, ligated with T4 DNA ligase (Thermo Scientific), and treated with *DpnI*. Plasmid assembly and cloning were performed according to EasyClone. For gene deletions, DNA fragments of 400–600 bp up- and downstream of the target gene and the KanMX cassette were amplified from the Yeast Knockout (YKO) collection (Wach et al. 1994, Winzeler et al. 1999, Giaever et al. 2002). All constructed plasmids were verified by Sanger sequencing (Eurofins Scientific SE). Yeast transformations were performed using a lithium acetate-based protocol as previously described (Gietz and Schiestl 2007). Transformants were selected on YPD plates containing 250 mg/l of nourseothricin (Jena Bioscience, AB-101) for gRNA selection or 200 mg/l G418 (Sigma Aldrich) for selection of deletion mutants.

### *p*-Coumaric acid and β-carotene cultivations

Cultivation for compound production was carried out in Mineral and Feed-In-Time (FIT) medium (Enpresso GmbH). Mineral medium (pH 6.0) was prepared as previously described (Jensen et al. 2014), containing 7.5 g/l (NH_4_)_2_SO_4_, 14.4 g/l KH_2_PO_4_, 0.5 g/l MgSO_4_·7H_2_O, 20 g/l D-glucose, 2 ml/l trace metals solution, and 1 ml/l vitamins. FIT medium was prepared as minimial medium but with 80 g/l of polysaccharide (Enpresso GmbH) and 1% of enzyme (Enpresso GmbH). For preculture preparation, three single colonies were inoculated from fresh plates in mineral medium in 24-well plates with air-penetrable lid (EnzyScreen, NL) and grown for 18 h at 30°C and 300 rpm agitation at 5 cm orbit cast. The preculture was inoculated into 3 ml fresh mineral medium at an optical density at 600 nm (OD_600_) of 0.1 in new 24-well plates. The plates were incubated for 72 h at 30°C with 300 rpm agitation. After 72 h of cultivation, the final OD_600_ was measured using a NanoPhotometer (Implen GmbH, Germany). For β-carotene, the cultivations were made in YP 8% D-glucose for intracellular extraction, and for extracellular cultivations were done in 10 ml glass tubes with a 10% layer of dodecane (Arnesen et al. 2020).

### Extraction and analytical methods

After 72 h cultivation, 1 ml of the cultivation broth was transferred into a 2-ml microtube (Sarstedt) for carotenoids extraction. For intracellular extraction, the sample was centrifuged at 10,000 *g* for 5 min and the supernatant removed. To each tube, 500 µl of 0.75 mm glass beads were added. A volume of 1 ml of ethyl acetate supplemented with 0.01% 3,5-di-tert-4-butylhydroxy toluene (BHT) was also added to each tube. Cells were disrupted using a Precellys®24 homogenizer (Bertin Corp.) in four cycles of 5500 rpm for 20 s. After disruption, the cells were centrifuged for 5 min at 10,000 *g* and the extract was subjected to HPLC analysis. For cell dry weight measurement 2 ml microtube (Sartedt) was dried overnight (ON) at 60°C. After 12–14 h, they were placed in a desiccator, and the weight was measured using an analytical balance. After 72 h of cultivation, 1 ml of sample was transferred to the preweighted tubes. Samples were centrifuged, and pellets were washed and dried in a 60°C incubator. Dry weight was measured after 96 h. For HPLC measurements, 100 μl of ethyl acetate extract from 1 ml culture was evaporated in a rotatory evaporator and dry extracts were redissolved in 1 ml 99% ethanol with 0.01% BHT. For extracellular concentration, 1 ml of culture was centrifuged, and the supernatant was mixed with 100 μl of dodecane. The mixture was centrifugated, and the dodecane layer was extracted and evaporated following the same procedure as the intracellular samples. Extracts were then analyzed by HPLC (Thermo Fisher Scientific, Waltham, USA) equipped with a Discovery HS F5 150 mm × 2.1 mm column (particle size 3 mm). The column oven temperature was set at 30°C. All organic solvents were HPLC grade (Sigma Aldrich). The flow rate was set at 0.7 ml/min with an initial solvent composition of 10 mM ammonium formate (pH 3, adjusted with formic acid; solvent A) and acetonitrile (solvent B; 3:1) until minute 2.0. Solvent composition was then changed following a linear gradient until % A = 10.0 and % B = 90.0 at 4.0 min. This solvent composition was kept until 10.5 min when the solvent was returned to the initial conditions, and the column was re-equilibrated until 13.5 min. The injection volume was 10 μl. Peaks were identified by comparison to the prepared standards, and integration of the peak areas was used to quantify carotenoids from obtained standard curves using the software Chromeleon7 (Thermo Fisher Scientific). β-Carotene had a retention time of 7.6 min and absorbance at 450 nm.

From the *p*-coumaric acid producer strains, 1 ml samples were mixed with an equal volume of absolute ethanol and centrifuged at 15,000 *g* for 5 min for extracellular quantification. The supernatant was stored at −20°C until analyzed. For intracellular quantification, 1 ml samples were centrifuged at 3000 *g* for 5 min, removed the supernatant, and washed the cells twice with water. 500 µl of 212–300 μm glass beads and 1 ml of 50% v/v ethanol were added, and cells were disrupted using a Precellys®24 homogenizer (Bertin Corp.) in four cycles of 5500 rpm for 20 s. Samples were centrifuged at 15,000 *g* for 5 min, and the supernatant was used for quantification. A volume of 5 μl of the sample was injected for the quantification. The HPLC system was equipped with a Discovery HS F5 150 mm × 2.1 mm column, particle size 3 μm (Supelco), and a DAD-3000 UV/Vis detector (Dionex). The column oven temperature was set at 30°C, and the flow rate to 0.7 ml/min. Solvent A was 10 mM ammonium formate (pH 3.0, adjusted by formic acid). Solvent B was acetonitrile. Solvent composition was initially A = 95.0%, and B = 5.0%, which was kept until 0.5 min. Then, solvent composition was changed following a linear gradient until A = 40.0%, and B = 60.0% at 7.0 min. These conditions were kept constant for 2.5 min (7.0–9.5 min). The solvent composition was returned linearly to the initial conditions (A = 95.0% and B = 5.0%) at 9.6 min and remained unchanged until the end of the run (9.6–12 min). *p*-Coumaric acid was detected at a retention time of 4.7 min, using the absorbance at 277 nm for the quantification.

### Caffeine and CFW spot and fluorescence assays

For the spot assays, cells were grown ON at 30°C in YED medium and its OD_600_ normalized to 0.1. 5 μl of cell suspensions were spotted in serial 10-fold dilution on YED, YED +12 mM caffeine, and YED +50 μg/ml CFW. Pictures were taken after 2 days at 30°C.

The fluorescence study was performed as described by de Groot et al.(2001). Cells from an ON culture were inoculated 1:10 in fresh YED medium and incubated for 5 h at 37°C. From each culture, a 200 μl sample was taken for centrifugation (1 min) and the supernatant was removed. Cells were resuspended in 50 ml of a 10 mg/ml solution of Calcofluor white (Fluorescent Brightener 28, Sigma Aldrich) and observed using a Leica DM4000B fluorescence microscope with Hg lamp, an appropriate set of filters (UV to blue for excitation and blue to green for emission) and a 100× immersion objective.

### RT-qPCR of *ECM33*

ON cultures in YP 8% D-glucose for the β-carotene production strains and FIT media for the *p*-CA production strains were inoculated to OD_600_ 0.1 and cultured for 16 h at 30°C. Cells were homogenized with TRI reagent (Sigma Aldrich) and the RNA was extracted with Direct-zol^TM^ RNA MiniprepPlus (Zymo Research), including a DNase I treatment. The RNA concentration and quality were determined using a NanoPhotometer (Implen GmbH). Reverse transcriptomics to cDNA was performed with High Capacity cDNA Reverse Transcription Kit (Thermo Fisher Scientific). mRNA expression levels were determined by qPCR. 2x SensiFAST SYBR® Lo-ROX mix (Nordic BioSite) was used for all qPCR reactions, consisting of 5 µl of master mix, 0.03 µl of each primer (100 µM; see Table S4, Supporting Information) and 5 µl of 20–25 ng of cDNA. All qPCR reactions followed the same thermocycler program that consisted of an initial 3 min step at 98°C, followed by 40 cycles of 98°C for 10 s, 60°C for 15 s, and 72°C for 30 s, along with a final melting curve consisting of a single cycle of 98°C for 5 s, 60°C for 1 min, and 98°C for 5 s. All samples were run in duplicate. All qPCR runs were performed on the Roche LightCycler® 480 Real-time PCR System in LightCycle® 480 Multiwell Plate 384, clear plates (Roche). Expression of *ECM33* was calculated based on the 2-ΔΔCT method (Rao et al. 2013) and normalized to the house-keeping gene *ALG9* (Teste et al. 2009).

### Electron microscopy

Cultures were fixed for 24–26 h in 2% paraformaldehyde and 1.5% glutaraldehyde in 0.05 M-sodium cacodylate buffer (pH 7.2) at 4°C as descriped by (Glauert 1975). The centrifuged cells were washed with saline and postfixed in 1% potassium permanganate for 90 min at 4°C. The cells were pelleted at low speed, washed with distilled water and briefly sonicated to remove fixation precipitates, washed again with water, dehydrated and embedded in Spurr’s low viscosity embedding resin (Taab Laboratories Equipment Ltd, Reading). Thin sections (120) nm were stained with uranyl acetate and with lead citrate and examined with a Thermo Fisher Tecnai T20 G2 equipped with a TVIPS XF416 CCD camera.

## Results

### Response of cell wall-engineered yeasts to cell wall stressors

**Figure 1.**
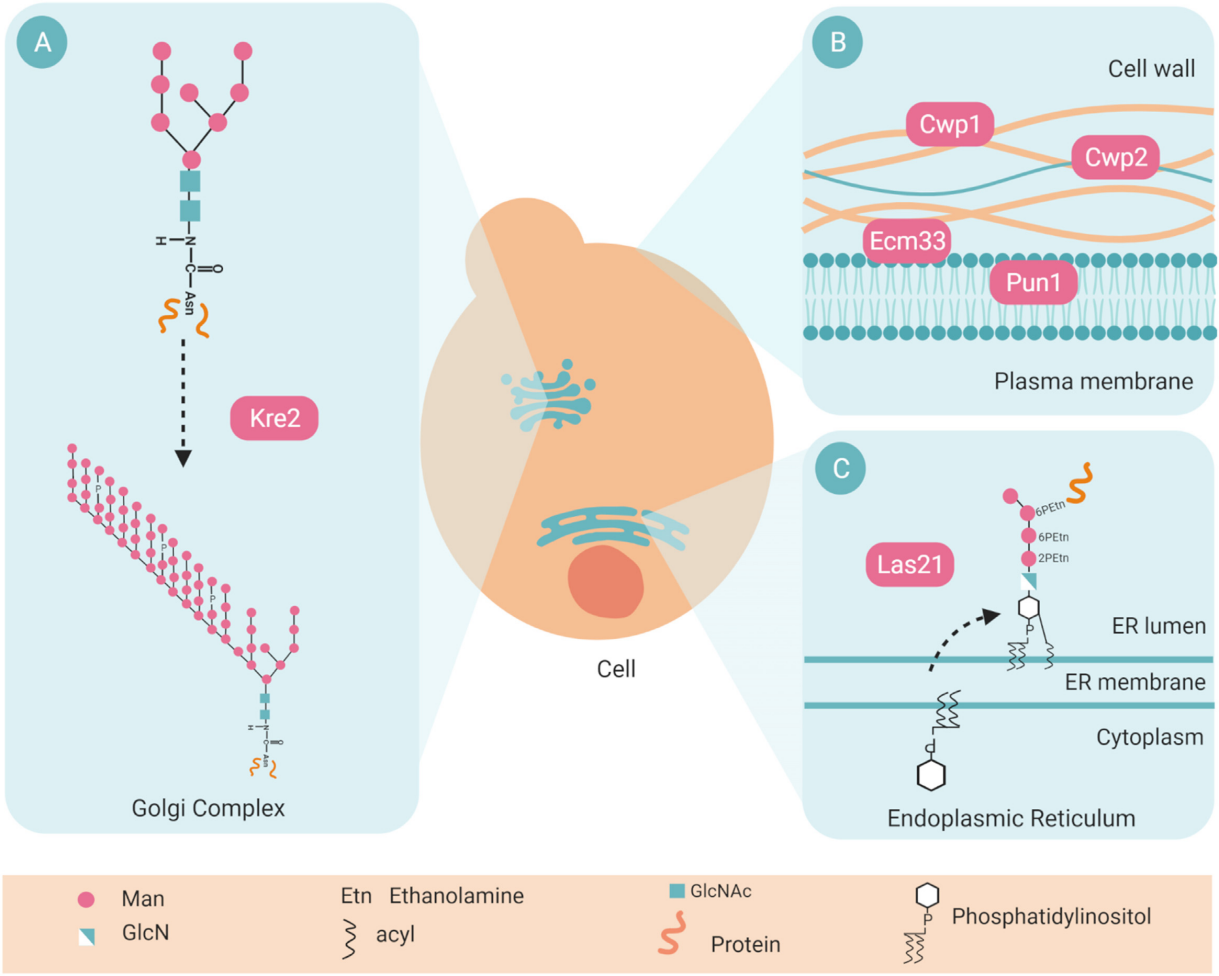
Localization and function of the proteins subject of the study. (A) Kre2p is an alpha1,2-mannosyltransferase of the Golgi apparatus that transfers an alpha-D-mannosyl residue from GDP-mannose into N- or O-linked oligosaccharides attached to proteins, forming an alpha-(1→2)-D-mannosyl-D-mannose linkage. (B) Ecm33p, Cwp1p, and Cwp2p are located in the cell wall, and Pun1p localizes to the plasma membrane. All are involved in CWI. (C) Las21p is a mannose–ethanolamine phosphotransferase involved in synthesizing GPI in the endoplasmic reticulum membrane. It catalyzes the addition of a side chain ethanolamine phosphate to the alpha1,6-linked second mannose residue of the GPI lipid precursor. Figure created with BioRender.com.

For this study, we selected six nonessential genes (*KRE2*,*CWP1*,*CWP2*,*ECM33*,*PUN1*, and *LAS21*) involved in CWI, cell wall biogenesis, degradation, and remodeling (Fig. 1). Kre2p contributes both to the elongation of the mannan outer chains in the N-linked glycosylation pathway and to the synthesis of O-linked oligosaccharides as part of the O-mannosylation pathway in the Golgi affecting CWPs modifications (Orlean 2012). Cwp1p and Cwp2p are major cell wall constituents, where they attach with a GPI-dependent linkage (Van der Vaart et al. 1995). Ecm33p is another GPI protein. The deletion of *ECM33* results in a disorganized and weak cell wall, activating the CWI pathway (Pardo et al. 2004). Pun1p is a plasma membrane protein induced upon metal ion stress and affects the response to nitrogen stress (Hosiner et al. 2011, Atanasova et al. 2018). Lastly, Las21p adds a side chain of GPI required to attach GPI-proteins to the cell wall (Benachour et al. 1999, Tohe and Oguchi 1999).

The compounds used in this study are β-carotene and *p*-coumaric acid. β-Carotene is a red–orange tetraterpenoid and is the precursor of Vitamin A (Krinsky and Johnson 2005). β-Carotene has antioxidant activity against reactive oxygen species (Young and Lowe 2001). *p*-Coumaric acid is a hydroxycinnamic acid having antioxidant, inflammatory, and antimicrobial properties (Luceri et al. 2007, Lou et al. 2012). Moreover, *p*-coumaric acid is a precursor for nutraceutical and pharmaceutical molecules (Călinoiu and Vodnar 2018, Lv et al. 2012).

We overexpressed or deleted the six selected cell wall-related genes in the strain producing *p*-coumaric acid (ST10283) or β-carotene (ST8939) (Milne et al. 2020). The overexpression was achieved by integrating a second copy of the gene under the native promoter or under the strong constitutive *TEF1* promoter (Kitamoto et al. 1998). We constructed a total of 36 cell wall-engineered strains. We evaluated the CWI by exposing the cell wall mutants to the two cell wall stressors, caffeine and calcofluor white (CFW) in a spot assay.

Caffeine has multiple intracellular targets, including induction of the CWI pathway, by activation of the MAP kinase Slt2 (Martín et al. 2000). CFW binds to nascent chitin chains, thereby weakening the cell wall (Ram and Klis 2006). The two compounds represent two routes of activating the CWI pathway. A permeable cell wall results in increased sensitivity to environmental stress and ultimately to cell death.

**Figure 2.**
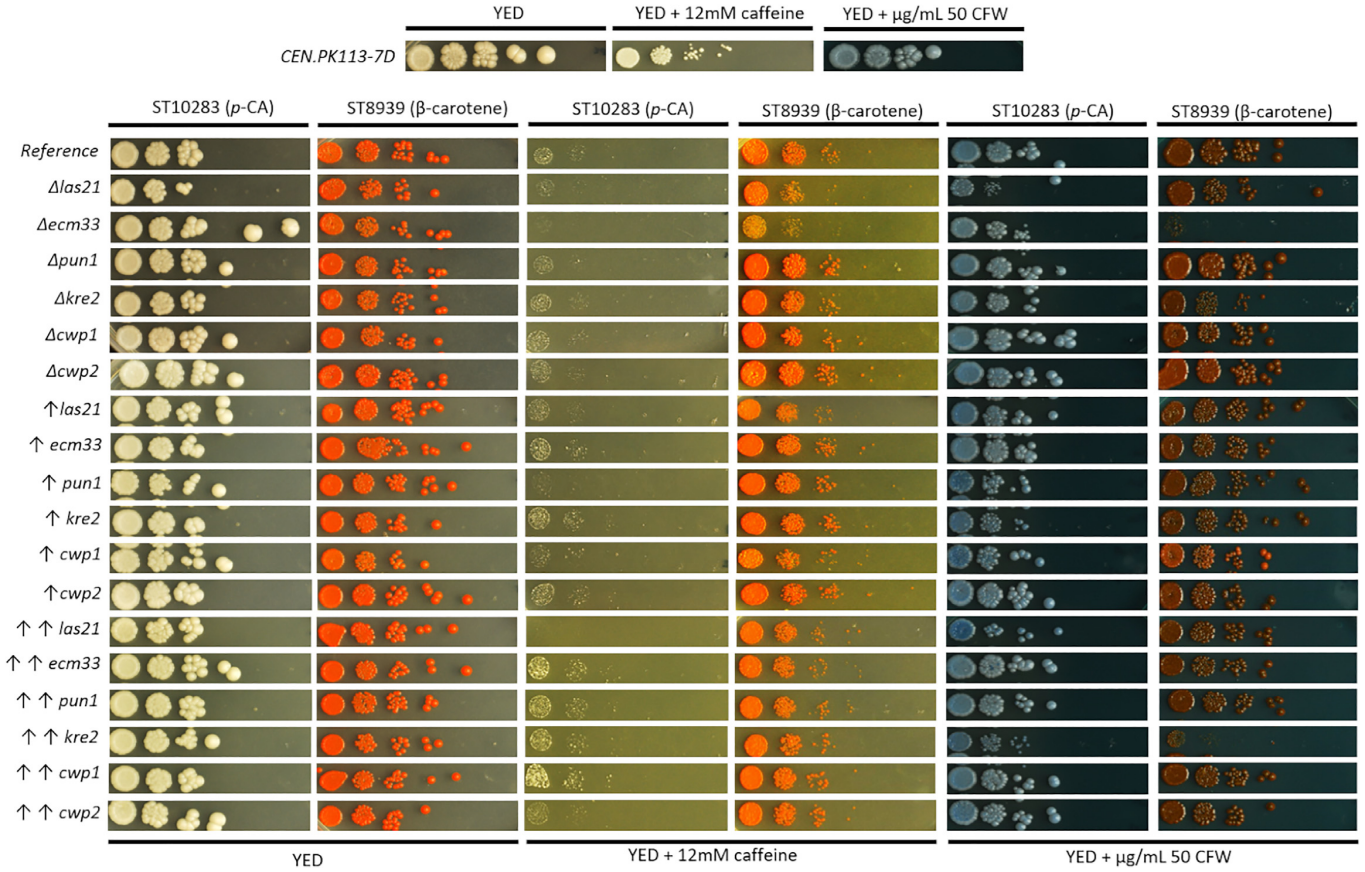
Spot assays in caffeine and CFW. Knockouts and upregulations with native and TEF1 promoter were spotted in serial 10-fold dilution on YED plates, YED with caffeine 12 mM and YED with CFW 50 μg/ml. Cells were grown ON at 30°C in YED medium and its OD_600_ normalized to 0.1, in 10 times serial dilutions, before spotting. After spotting on the indicated plates, they were incubated ON at 30°C. CEN.PK113-7D is the wild-type strain and the reference strains are ST10283 for *p*-coumaric acid and ST8939 for β-carotene, which were used as control. The symbol ↑ represents upregulation with a double copy integration with the native promoter, and ↑↑ with *TEF1* promoter.

Of the tested mutations, the most pronounced growth defects were found in the *Δlas21* and *Δecm33* knockouts in both the *p*-coumaric and β-carotene producing strains (Fig. 2). This result is in agreement with previous studies where *Δecm33* was also found to be caffeine-hypersensitive compared to a wild-type strain (de Groot et al. 2001, Pardo et al. 2004). Both of the knockouts presented increased amounts of cell wall components in the growth medium, a phenotype related to a weakened cell wall.

Curiously, overexpression of *LAS21* completely abolished the growth of *p*-coumaric strain in the presence of caffeine. Overexpression of *ECM33*, *PUN1, KRE2*, and *CWP1* genes from the strong promoter *TEF1* in the *p*-coumaric acid strain slightly improved tolerance to caffeine. Both production strains with upregulation of *KRE2*, especially from the *TEF1* promoter, showed increased sensitivity to CFW, while on caffeine, the response was comparable to the reference strain.

The increased sensitivity indicates that the upregulation of *LAS21* and *KRE2* may activate the CWI pathway, depending on the produced compound. Increased sensitivity to cell wall stressors was previously shown for *CWP2*, where both the deletion and upregulation activated the CWI pathways (Wentz and Shusta 2007). Interestingly, neither of the *CWP2* mutants tested here showed a change in sensitivity in the two tested stress conditions, in either of the production strains. The lack of response for the *CWP2* mutants could indicate an interplay between the CWI pathway and the produced compounds, as was also observed for squalene (Valachovic et al. 2016).

We observed differences in response for the mutants depending on which compound was produced. For example, the *p*-coumaric acid producer strain showed increased sensitivity to caffeine compared to the nonproducing background. In contrast, the β-carotene producing strain showed similar sensitivity to caffeine as the wild-type strain.

While *p*-coumaric acid is water soluble, β-carotene is a lipophilic compound shown to associate with cellular membranes and lipid droplets (Bu et al. 2022). The association of β-carotene with cellular membranes can activate the CWI pathway directly or alter the fluidity of the membranes. We observed no growth phenotype for the β-carotene producer strain compared to the CEN.PK background, indicating the activation of the CWI pathway is kept in balance. We hypothesize that the combination of cell wall stressors and cell wall deletion mutants can shift this balance, creating a conditional sensitivity.

This conditional sensitivity appears to be most pronounced with CFW, which also is the cell wall stressor that most directly decreases the rigidity of the cell wall.

The production of *p*-coumaric acid has been shown to cause the downregulation of amino acid and sugar transporter proteins (Rodriguez et al. 2017). Caffeine has many intracellular targets, including the TOR 1/2 complex [(Kuranda et al. 2006), reviewed in (Ruta and Farcasanu 2020)]. The TOR complex regulates the activity of many intracellular targets, including amino acid transporters. The TOR complex alters the phosphorylation pattern on the ubiquitin ligase adapter Art1, thereby halting or inducing endocytosis of the transporter proteins targeted by Art1 (MacGurn et al. 2011).

The TOR complex also targets components of the MCC, plasma membrane compartments that shelter specific transporter proteins (Stradalova et al. 2012, Gournas et al. 2018). Our results would indicate that the downregulation of amino acid transporters in the *p*-coumaric acid producer strain is further challenged by the addition of caffeine, creating a conditional growth phenotype. The effect of caffeine is possible through the TOR complex and additional downregulation of amino acid transporters.

### Influence of cell wall engineering on *p*-coumaric acid and β-carotene production

**Figure 3.**
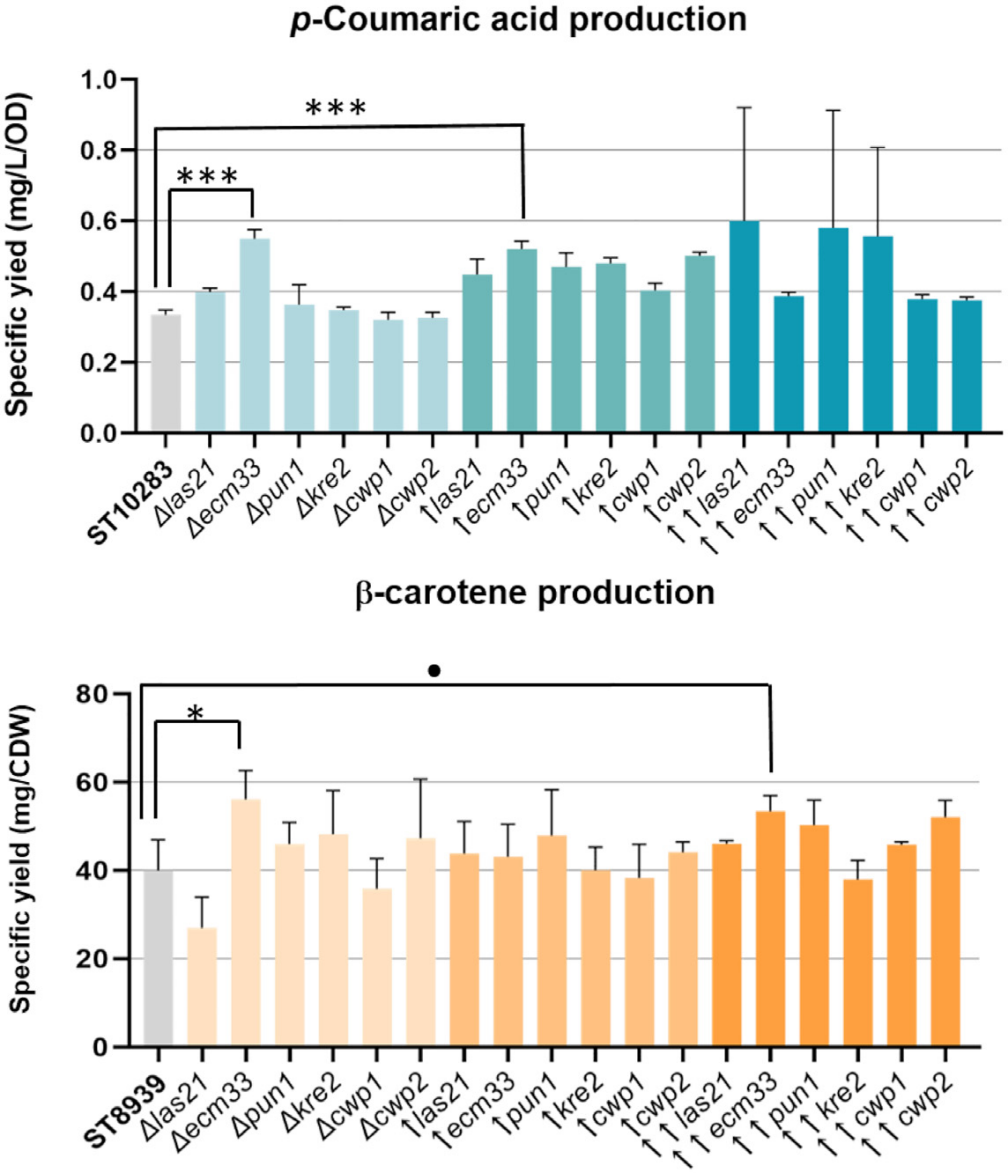
Yields of *p*-coumaric acid and β-carotene in cell wall-engineered *S. cerevisiae* strains. Cultivations were carried out for 72 h in 24 deep-well plates containing FIT medium with 60 g/l of polysaccharide or YP 8% D-glucose. Extracellular content for *p*-coumaric acid and intracellular for β-carotene were subjected to HPLC analysis. Error bars represent the standard deviation from three biological replicates. The symbol ↑ represents upregulation with a double copy integration with the native promoter and ↑↑ represents *TEF1* promoter. Statistical analysis was performed using Student's *t*-test (two-tailed; * = *P* ≤ .05; ** = *P* ≤ .01; *** = *P* ≤ .001; ● = 0.05898; two-sample unequal variance). A version of the figure with the individual data points indicated can be found in Figure S1.1 (Supporting Information; *p*-coumaric acid) and Figure S2.2 (Supporting Information; β-carotene).

To study the influence of the cell wall on compound production, we cultivated the cell wall mutants in liquid medium and measured the production of the target metabolites. The only positive effect was observed for *ecm33∆* deletion in the the *p*-coumaric acid strain, where the specific yield of *p*-coumaric acid increased by 40% and for ECM33 overexpression from the *TEF1* promoter, resulting in 36.5% increase (Fig. 3). The intracellular content of *p*-coumaric acid in all the strains was very low, indicating that secretion is not a limiting factor for *p-*coumaric acid production [Figure S3 (Supporting Information), a figure with the individual data points is available in Figure S3.1 (Supporting Information)].

When the same cell wall mutants were tested for production in the β-carotene strain reference strain, the deletion or upregulation of *ECM33* also resulted in significantly higher product content. However, we only observed the increase in titer with *ECM33* expressed from the strong promoter *TEF1*. We observed no increase in β-carotene titer when *ECM33* was expressed from an extra copy of its native promoter. We also quantified the extracellular concentration of β-carotene. We detected almost no secretion of β-carotene in the parental production strain, and no significant effect was observed in the mutants [Figure S4 (Supporting Information), a figure with the individual data points is available in Figure S4.1 (Supporting Information)].

Moreover, we did not observe any effect on production or secretion from the *KRE2* and *LAS21* mutants, though both mutants showed increased sensitivity towards the cell wall stressors.

### The expression of *ECM33* is influenced by the production of *p*-coumaric acid and β-carotene

When we assessed the *p*-coumaric acid and β-carotene production in cell wall mutant strains, we observed a difference between the mutants expressing *ECM33* from the native promoter compared to the TEF1 promoter depending on the production background. We performed a RT-qPCR on *ECM33* cDNA in the *ECM33* mutants in both production backgrounds to investigate this phenomenon.

**Figure 4.**
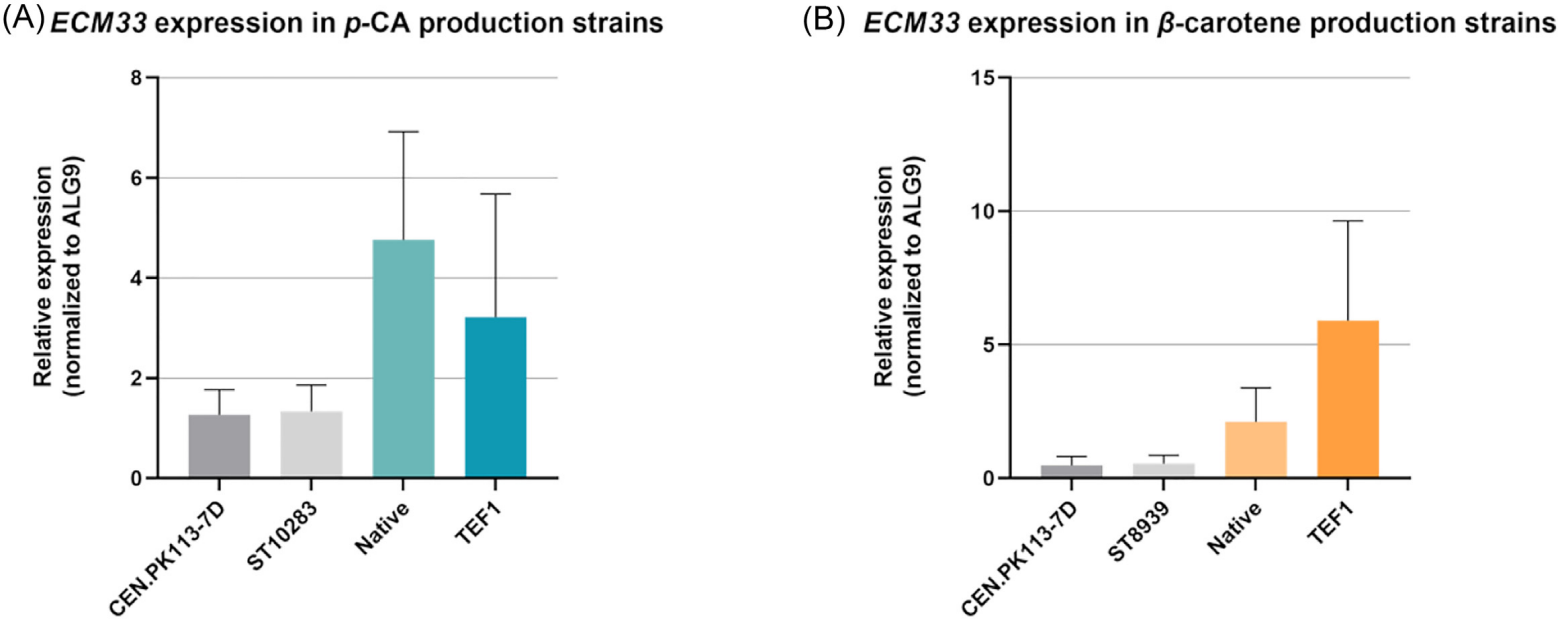
*ECM33* expression in *p*-coumaric acid and β-carotene production strains. Expression of ECM33 was measured by RT-qPCR after 16 h of growth. The expression values are normalized to the house-keeping gene ALG9. (A) Relative expression of ECM33 in the *wt* strain CEN.PK113-7D, p-CA reference strain, strain overexpressing *ECM33* from its native promoter or from TEF1 promoter (B) Relative expression of *ECM33* in the *wt* strain CEN.PK113-7D, β-carotene reference strain, and strain overexpressing *ECM33* with its native promoter and with TEF1 promoter

To our surprise, the expression of *ECM33* from the native promoter greatly depended on which production background harbored the mutant, while the *ECM33* cDNA levels showed less dependency on the production background when expressed from pTEF1 (Fig. 4). The mutant strains with more *ECM33* cDNA relative to the housekeeping gene *ALG9*, correlates with the mutant strains with higher production yield, suggesting overexpression of *ECM33* causes higher production.

### Chitin deposition patterns are different in *ECM33* deletion and overexpression strains

Our previous experiments suggested a correlation between an efficient overexpression of *ECM33* and increased production yield. Therefore, we sought to investigate if the overexpression of *ECM33* influenced the morphology of the cell wall, which could explain the increase in yield.

Chitin content influences the cell wall rigidity. An *ecm33∆* mutant was previously shown to increase chitin deposition in the cell wall, as its deletion upregulates the chitin synthase *CHS3* (de Groot et al. 2001, Zhang et al. 2018). To determine if the *ECM33* mutants strengthen the cell wall through increased chitin deposition, we analyzed the chitin deposition by CFW staining and observed it with fluorescence microscopy.

We evaluated the chitin deposition in both the β-carotene and *p*-coumaric acid production strains to determine any differences in chitin deposition related to the small molecule production.

**Figure 5.**
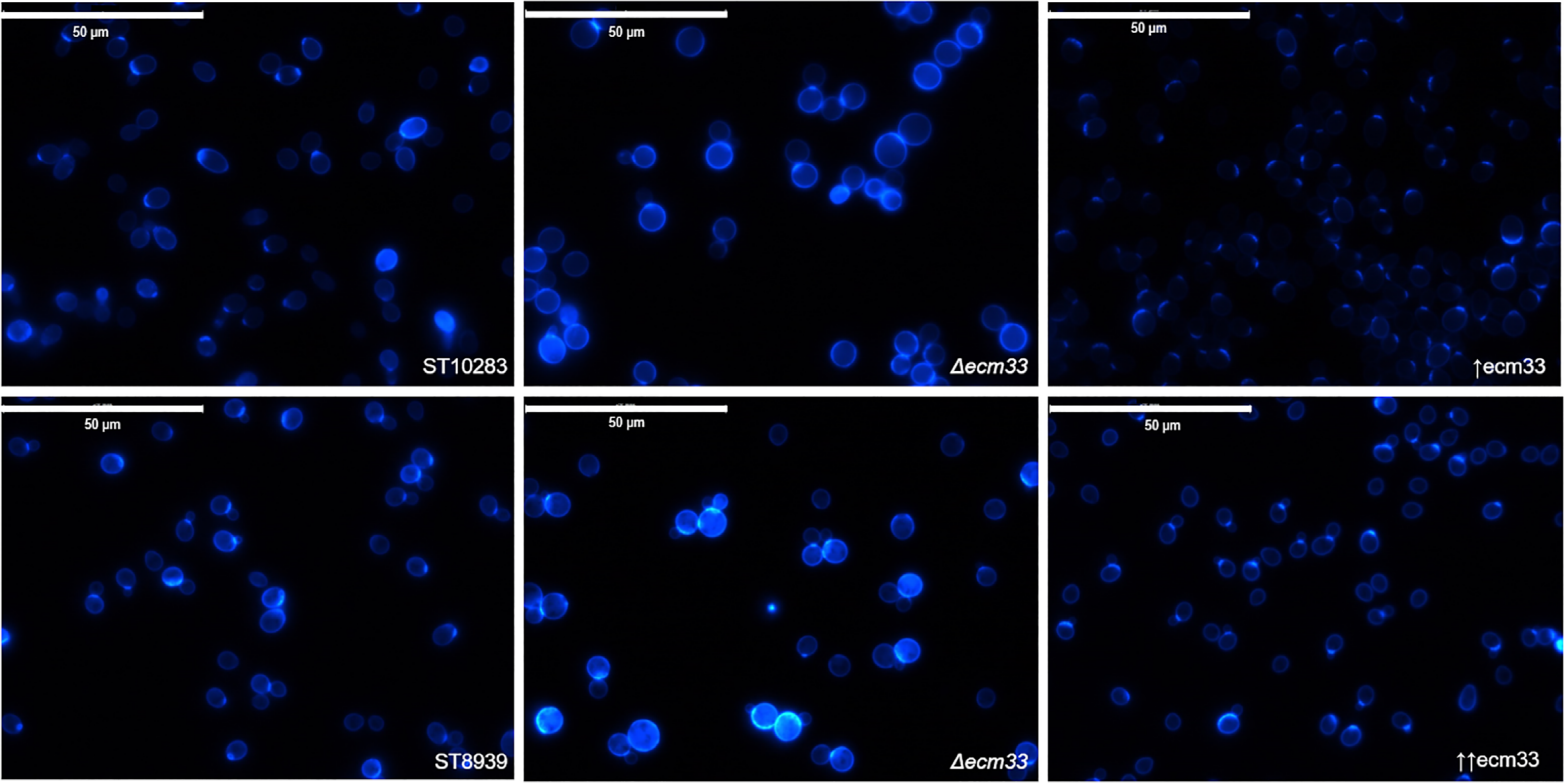
Chitin deposition and morphology in cell wall engineered *S. cerevisiae* strains. Cells were grown at 37°C for 5 h and subsequently stained with calcofluor white. The bars indicate 50 µm. The symbol ↑ represents upregulation with a double copy integration with the native promoter and ↑↑represents *TEF1* promoter.

The chitin content was predominantly rich in budding scars in both the parental production strains. This is in agreement with previous observations (Schekman and Brawley 1979). There was no visual difference in chitin content between the two production strains. Also, the *ECM33* overexpression mutants in the two production backgrounds behaved similarly. All the *ECM33* mutant strains presented a more homogeneous distribution alongside the cell wall. The *ecm33∆* showed higher fluorescence than their parental counterparts, indicating a higher chitin content in the cell wall (Fig. 5).

Furthermore, the *ecm33∆* mutants presented an abnormal morphology characterized by a swollen shape. Both of the producer cells, and the producer cells with the upregulated *ECM33* had a normal morphology.

The chitin abundance is similar in both upregulation strains, probably because despite having different promoters, there is gene upregulation in both of them resulting in the same phenotype. While the *ecm33∆* has been shown to activate the CWI pathway and increase chitin deposition, this is not the case for the *ECM33* upregulated strains. The *ECM33* upregulated strains have decreased chitin content, suggesting an inhibition of the CWI pathway. Moreover, the difference in chitin deposition indicates that the observed increase in production yield in the *ECM33* overexpressing mutants does not arise from a chitin-strengthened cell wall.

### The upregulation of *ECM33* makes the cell wall thicker

Previous studies have observed a disorganized cell wall structure in an *ecm33∆* mutant (Pardo et al. 2004), suggesting the increase in chitin content in the *ecm33∆* mutant correlates with a weakened cell wall. No studies have reported the effect of *ECM33* overexpression on the cell wall morphology. We used electron microscopy to evaluate any changes in cell wall morphology in the *ecm33∆* knockout and *ECM33* overexpression mutants.

**Figure 6.**
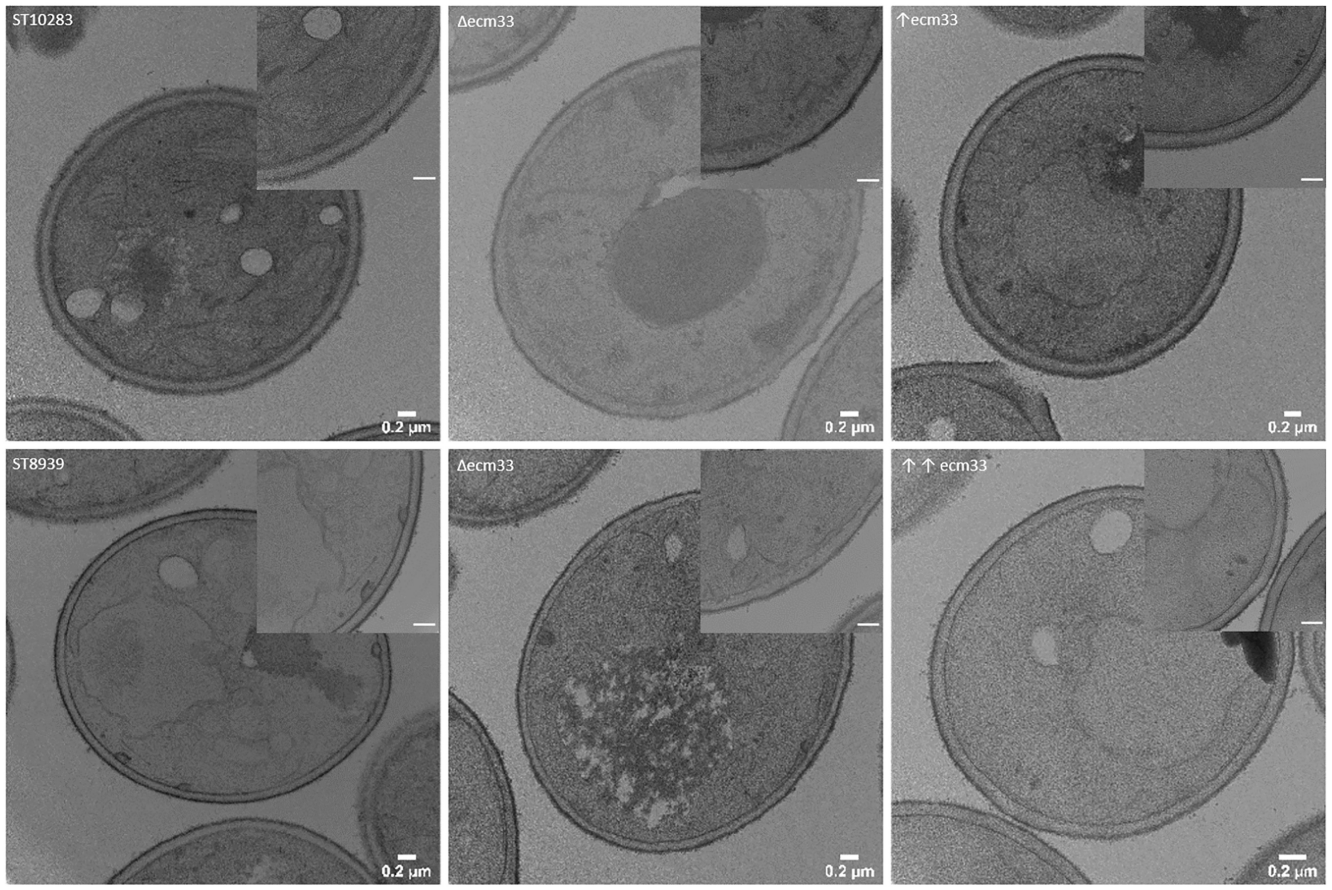
TEM images of *ECM33* mutants in two different engineered *S. cerevisiae* background strains. Cells were grown to OD 1 and processed for visualization as described in the materials and methods. The upper row of images correspond to the *p*-coumaric acid producer strains. Bottom row of images correspond to the β-carotene producer strains. The details of the cell wall are shown with a 0.2-um scale bar. The symbol ↑ represents upregulation with a double copy integration with the native promoter and ↑↑ represents *TEF1* promoter.

Both the β-carotene and *p*-coumaric acid producer strains showed regular and organized outer mannoprotein layer, indicating that the produced molecules do not cause any observable alteration to the cell wall (Fig. 6). The cell wall of the *ecm33∆* mutants was thinner and more irregular than the producer strains without mutations, to the extent where the mannoprotein layer is markedly thinner or almost nonvisible in some regions. The upregulation of *ECM33* in both the β-carotene and *p*-coumaric acid producer strains resulted in a thicker and more homogeneous cell wall than their parental strains. The cell wall defect observed in the *ecm33∆* mutants is likely due to an incomplete mannoprotein layer with an incomplete assembly of 1,6-β-glucan-linked proteins to the cell wall.

The thicker cell wall observed in the strains with an upregulation of *ECM33* could likewise be due to an additional 1,6-β-glucan-linkage of proteins to the cell wall.

## Discussion

This study explored the connection between the cell wall structure, cell wall stress, and production and secretion of small compounds. We deleted and overexpressed six cell wall-related genes to modulate the cell wall (*LAS21*, *KRE2*, *ECM33*, *PUN1*, *CWP1*, and *CWP2*).

In our initial evaluation, we used the cell wall stressors caffeine and CFW to assess the sensitivity of the cell wall mutants in two different production backgrounds. Caffeine activates the CWI pathway, while CFW is hypothesized to bind to chitin and inhibit its correct assembly, which counteracts the CWI pathway response and amplifies the effect of cell wall deletions (Ram et al. 1994, Ram and Klis 2006).

*Las21∆* and *ecm33∆* showed increased sensitivity to both cell wall stressors, while the upregulation of *LAS21* from the strong *TEF1* promoter also increased sensitivity to caffeine. Deletion of *LAS21* and *ECM33* has previously been shown to cause increased sensitivity to cell wall stressors (Tohe and Oguchi 1999, de Groot et al. 2001, Fujita et al. 2004). In an *ecm33∆* mutant, the CWI pathway is activated by upregulation of the chitin synthase Chs3p, the mitogen-activated-protein kinase Hog1p, and the serine/threonine-protein kinase Slt2p (Pardo et al. 2004).

Las21p is required to add a side chain to the GPI core structure, and its deletion shows increased levels of cell wall components in the supernatant (Benachour et al. 1999, de Groot et al. 2001). The upregulation of *LAS21*, especially from the *TEF1* promoter, showed increased sensitivity toward caffeine. The increase in sensitivity can be due to a hyperactivation of the CWI pathway, which was previously shown to lead to growth defects (Jiménez-Gutiérrez et al. 2020). The increased sensitivity was also observed for *LAS21* on CFW, though only when expressed from the *TEF1* promoter in the *p*-coumaric acid-producing background. The difference in sensitivity on caffeine and CFW for the *LAS21* overexpressing mutants points toward the activation of the CWI pathway. While on caffeine, the CWI pathway is overactivated, leading to a growth phenotype, CFW counteracts the overactivation of the CWI. It is very much a balancing act, as the strong overexpression of *LAS21* in the *p*-coumaric acid-producing background seems to overcome the action of the CFW and show a growth phenotype.

In addition, *kre2∆* and the upregulation of *KRE2* from the *TEF1* promoter showed increased sensitivity to CFW in both production backgrounds. The increased sensitivity on CFW would suggest activation of the CWI pathway in a manner similar to *LAS21*.

When we quantified the production of *p*-coumaric acid and β-carotene in the cell wall mutants, we observed a significant increase in yield in the *p*-coumaric acid producer strain in the *ecm33∆* background. Deleting *ECM33* has previously been shown to increase the squalene production in *S. cerevisiae* (Son et al. 2020), as well as the fermentation capacity in a wine yeast (Zhang et al. 2018), potentially through an increased chitin deposition strengthening the cell wall, making it more robust in a fermentation setup.

In our study, also overexpression of *ECM33* resulted in better production. The improved production in the *ECM33* overexpression strain agrees with our initial characterization of *ECM33* on caffeine and CFW. In our characterization of the cell wall mutants, overexpression of *ECM33* also resulted in an increased sensitivity to caffeine and CFW. However, we only observed an improvement in production for the *ECM33* mutants, not any of the other cell wall mutants that showed an altered sensitivity to caffeine or CFW in our initial characterization. The effect of *ECM33* on production is thus due to a unique target downstream of *ECM33* rather than the general CWI pathway.

To our surprise, we did not observe a significant difference in secretion of either *p*-coumaric acid or β-carotene in any of the cell wall mutants, suggesting the diffusion of *p*-coumaric acid or β-carotene through the cell wall at the current production level is not a limiting factor. Interestingly, in the *ECM33* mutant strains with increased production, the extracellular fraction of *p*-coumaric acid or β-carotene has not increased, suggesting that the passage through the plasma membrane or the cell wall could become a limiting factor at higher production levels. The improvement in production in the *ECM33* mutants is likely due to an improvement in the cell wall making the cells more robust.

While the *ecm33∆* and overexpression of *ECM33* showed the same phenotype in production and sensitivity toward cell wall stressors, the evaluation of the cell wall revealed a difference in the two mutant strains in both production backgrounds. The chitin content was increased in the *ecm33∆* strains when visualized with CFW staining, the cell wall appeared more irregular, and the plasma membrane was more diffuse when observed with TEM. The increase in chitin content agrees with de Groot et al. (2001). We hypothesize that the increased rigidity from chitin is needed as a compensation mechanism to counteract the more porous cell wall observed by TEM.

For the mutants with *ECM33* overexpressed, we observed a slight decrease in chitin content, while the cell wall appeared thicker when observed by TEM. The overexpression of *ECM33* has, thus the direct opposite effect on the chitin content and cell wall morphology compared to the deletion mutants. Rather than hyperactivating the CWI pathway, the *ECM33* overexpression mutants seem to counteract the CWI response. Further, we did not observe any growth defects in the *ECM33* overexpression mutants (Figures S1 and S2, Supporting Information), a phenotype typically observed in CWI hyperactivation.

Our results would suggest that the *ECM33* overexpression mutants inhibit the CWI pathway, potentially by inhibiting one of the CWI pathway proteins like the MAP kinase Slt2p. Pardo et al. (2004) previously showed that Slt2p is activated in an *ecm33∆* mutant. *ECM33* is also associated with efficient glucose uptake through the TOR complex (Umekawa et al. 2017). Moreover, caffeine has been shown to inhibit the TOR complex (Martin et al. 2015). If the overexpression of *ECM33* leads to an increase in glucose uptake and ATP production, this could explain the increased yield of *p*-coumaric acid and β-carotene we observed. Further experiments are needed to elucidate the molecular mechanism behind the yield enhancement by the *ECM33* overexpression.

## Conclusions

In this study, we showed that both the upregulation and deletion of the gene *ECM33* resulted in higher production yields of β-carotene and *p*-coumaric acid. The deletion of *ECM33* likely strengthened the cell wall, making the cells more robust for biotechnological production. The mechanism governing the yield increase in the *ECM33* overexpression mutants is yet unidentified. Compound secretion was not affected in any cell wall mutant strains suggesting the cell wall is not the determining factor for the secretion of small molecules at the current production levels. These findings broaden our knowledge on the cell wall and draws attention to the cell wall as a possible metabolic engineering target.

## Supplementary Material

foac037_Supplemental_files
