# Supplementary material for: Modulation of the cell wall protein Ecm33p in yeast Saccharomyces cerevisiae improves the production of small metabolites
Source: FEMS Yeast Res. 2022 Aug 3;22(1):foac037. doi: 10.1093/femsyr/foac037 (PMC9440718; doi:10.1093/femsyr/foac037)
Supplement: foac037_Supplemental_files [file foac037_supplemental_files.zip › Supplementary_Information_Final.docx]

**Supplementary information 1**

Strains, plasmids, biobricks, primers and DNA fragments used in this study

Table S1. Strains used in this study

| **Strain name** | **Genotype** | **Parent strain** | **Repair vector** | **Source** |
| --- | --- | --- | --- | --- |
| 1361 | *MATa his3Δ1 leu2Δ0 met15Δ0 ura3Δ0 Δlas21* | BY4741 |  | YKO Collection |
| 3215 | *MATa his3Δ1 leu2Δ0 met15Δ0 ura3Δ0 Δecm33* | BY4741 |  | YKO Collection |
| 5322 | *MATa his3Δ1 leu2Δ0 met15Δ0 ura3Δ0 Δpun1* | BY4741 |  | YKO Collection |
| 4945 | *MATa his3Δ1 leu2Δ0 met15Δ0 ura3Δ0 Δcwp1* | BY4741 |  | YKO Collection |
| 7026 | *MATa his3Δ1 leu2Δ0 met15Δ0 ura3Δ0 Δcwp2* | BY4741 |  | YKO Collection |
| 4317 | *MATa his3Δ1 leu2Δ0 met15Δ0 ura3Δ0 Δkre2* | BY4741 |  | YKO Collection |
| CEN.PK113-7D | *MATa URA3 HIS3 LEU2 TRP1 MAL2-8c SUC2* |  |  | (Entian & Kötter, 2007) |
| ST8939 | *MATa URA3 HIS3 LEU2 TRP1 MAL2-8c SUC2*  *X-4::XdCrtI-XdCrtYB XII-5::XdCrtE XI-3::XdCrtI-tHMG1*  *+ pCfB2312 (2µm cas9 KanMX)* | CEN.PK113-7D |  | (Milne et al., 2020) |
| ST9769 | *MATa URA3 HIS3 LEU2 TRP1 MAL2-8c SUC2*  *X-4::XdCrtI-XdCrtYB XII-5::XdCrtE XI-3::XdCrtI-tHMG1* | ST8939 | Cas9 removed | This study |
| ST9797 | *MATa URA3 HIS3 LEU2 TRP1 MAL2-8c SUC2*  *X-4::XdCrtI-XdCrtYB XII-5::XdCrtE XI-3::XdCrtI-tHMG3*  *Δpun1* | ST9769 | BB4528 | This study |
| ST9824 | *MATa URA3 HIS3 LEU2 TRP1 MAL2-8c SUC2*  *X-4::XdCrtI-XdCrtYB XII-5::XdCrtE XI-3::XdCrtI-tHMG1*  *Δlas21* | ST9769 | BB4526 | This study |
| ST9825 | *MATa URA3 HIS3 LEU2 TRP1 MAL2-8c SUC2*  *X-4::XdCrtI-XdCrtYB XII-5::XdCrtE XI-3::XdCrtI-tHMG2*  *Δecm33* | ST9769 | BB4527 | This study |
| ST10267 | *MATa URA3 HIS3 LEU2 TRP1 MAL2-8c SUC2*  *X-4::XdCrtI-XdCrtYB XII-5::XdCrtE XI-3::XdCrtI-tHMG6*  *Δkre2* | ST9769 | BB4800 | This study |
| ST10268 | *MATa URA3 HIS3 LEU2 TRP1 MAL2-8c SUC2*  *X-4::XdCrtI-XdCrtYB XII-5::XdCrtE XI-3::XdCrtI-tHMG4*  *Δcwp1* | ST9769 | BB4798 | This study |
| ST10279 | *MATa URA3 HIS3 LEU2 TRP1 MAL2-8c SUC2*  *X-4::XdCrtI-XdCrtYB XII-5::XdCrtE XI-3::XdCrtI-tHMG5*  *Δcwp2* | ST9769 | BB4799 | This study |
| ST10766 | X-4,, XdCrtI<-pTDH3-pTEF1->XdCrtYB, XII-5,, pPGK1->XdCrtE. XI-3,, XdCrtI<-pTDH3-pTEF1->tHMG1 XII-1:: Las21 | ST9769 | pCfB10313 | This study |
| ST10767 | X-4,, XdCrtI<-pTDH3-pTEF1->XdCrtYB, XII-5,, pPGK1->XdCrtE. XI-3,, XdCrtI<-pTDH3-pTEF1->tHMG1 XII-1:: Ecm33 | ST9769 | pCfB10314 | This study |
| ST10768 | XdCrtI<-pTDH3-pTEF1->XdCrtYB, XII-5,, pPGK1->XdCrtE. XI-3,, XdCrtI<-pTDH3-pTEF1->tHMG1 XII-1:: Pun1 | ST9769 | pCfB10315 | This study |
| ST10769 | X-4,, XdCrtI<-pTDH3-pTEF1->XdCrtYB, XII-5,, pPGK1->XdCrtE. XI-3,, XdCrtI<-pTDH3-pTEF1->tHMG1 XII-1:: Kre2 | ST9769 | pCfB10316 | This study |
| ST10770 | X-4,, XdCrtI<-pTDH3-pTEF1->XdCrtYB, XII-5,, pPGK1->XdCrtE. XI-3,, XdCrtI<-pTDH3-pTEF1->tHMG1 XII-1:: Cwp1 | ST9769 | pCfB10317 | This study |
| ST10771 | X-4,, XdCrtI<-pTDH3-pTEF1->XdCrtYB, XII-5,, pPGK1->XdCrtE. XI-3,, XdCrtI<-pTDH3-pTEF1->tHMG1 XII-1:: Cwp2 | ST9769 | pCfB10318 | This study |
| ST10772 | X-4,, XdCrtI<-pTDH3-pTEF1->XdCrtYB, XII-5,, pPGK1->XdCrtE. XI-3,, XdCrtI<-pTDH3-pTEF1->tHMG1 XII-1:: Las21 | ST9769 | pCfB10319 | This study |
| ST10773 | X-4,, XdCrtI<-pTDH3-pTEF1->XdCrtYB, XII-5,, pPGK1->XdCrtE. XI-3,, XdCrtI<-pTDH3-pTEF1->tHMG1 XII-1:: Ecm33 | ST9769 | pCfB10320 | This study |
| ST10774 | X-4,, XdCrtI<-pTDH3-pTEF1->XdCrtYB, XII-5,, pPGK1->XdCrtE. XI-3,, XdCrtI<-pTDH3-pTEF1->tHMG1 XII-1:: Pun1 | ST9769 | pCfB10321 | This study |
| ST10775 | X-4,, XdCrtI<-pTDH3-pTEF1->XdCrtYB, XII-5,, pPGK1->XdCrtE. XI-3,, XdCrtI<-pTDH3-pTEF1->tHMG1 XII-1:: Kre2 | ST9769 | pCfB10322 | This study |
| ST10776 | X-4,, XdCrtI<-pTDH3-pTEF1->XdCrtYB, XII-5,, pPGK1->XdCrtE. XI-3,, XdCrtI<-pTDH3-pTEF1->tHMG1 XII-1:: Cwp1 | ST9769 | pCfB10323 | This study |
| ST10777 | X-4,, XdCrtI<-pTDH3-pTEF1->XdCrtYB, XII-5,, pPGK1->XdCrtE. XI-3,, XdCrtI<-pTDH3-pTEF1->tHMG1 XII-1:: Cwp2 | ST9769 | pCfB10324 | This study |
| ST10283 | *Δpdc5, Δaro10, ↑FjTAL, ↑EcAroL, ΔGPP1, ↑Aro4*, ↑Aro7*, ↑Aro1, ↑Aro2, ↑Aro3, ↑PHA2 (2µm cas9 KanMX)* | CEN.PK113-7D |  | In-house |
| ST10575 | *Δpdc5, Δaro10, ↑FjTAL, ↑EcAroL, ΔGPP1, ↑Aro4*, ↑Aro7*, ↑Aro1, ↑Aro2, ↑Aro3, ↑PHA2* | ST10283 | Cas9 removed | This study |
| ST10521 | *Δpdc5, Δaro10, ↑FjTAL, ↑EcAroL, ΔGPP1, ↑Aro4*, ↑Aro7*, ↑Aro1, ↑Aro2, ↑Aro3, ↑PHA2 ΔLas21* | ST10575 | BB4526 | This study |
| ST10522 | *Δpdc5, Δaro10, ↑FjTAL, ↑EcAroL, ΔGPP1, ↑Aro4*, ↑Aro7*, ↑Aro1, ↑Aro2, ↑Aro3, ↑PHA2 Δecm33* | ST10575 | BB4527 | This study |
| ST10523 | *Δpdc5, Δaro10, ↑FjTAL, ↑EcAroL, ΔGPP1, ↑Aro4*, ↑Aro7*, ↑Aro1, ↑Aro2, ↑Aro3, ↑PHA2 Δpun1* | ST10575 | BB4528 | This study |
| ST10524 | *Δpdc5, Δaro10, ↑FjTAL, ↑EcAroL, ΔGPP1, ↑Aro4*, ↑Aro7*, ↑Aro1, ↑Aro2, ↑Aro3, ↑PHA2 Δkre2* | ST10575 | BB4800 | This study |
| ST10525 | *Δpdc5, Δaro10, ↑FjTAL, ↑EcAroL, ΔGPP1, ↑Aro4*, ↑Aro7*, ↑Aro1, ↑Aro2, ↑Aro3, ↑PHA2 Δcwp1* | ST10575 | BB4798 | This study |
| ST10526 | *Δpdc5, Δaro10, ↑FjTAL, ↑EcAroL, ΔGPP1, ↑Aro4*, ↑Aro7*, ↑Aro1, ↑Aro2, ↑Aro3, ↑PHA2 Δcwp2* | ST10575 | BB4799 | This study |
| ST10576 | *Δpdc5, Δaro10, ↑FjTAL, ↑EcAroL, ΔGPP1, ↑Aro4*, ↑Aro7*, ↑Aro1, ↑Aro2, ↑Aro3, ↑PHA2 XII-1:: Las21* | ST10575 | pCfB10313 | This study |
| ST10577 | *Δpdc5, Δaro10, ↑FjTAL, ↑EcAroL, ΔGPP1, ↑Aro4*, ↑Aro7*, ↑Aro1, ↑Aro2, ↑Aro3, ↑PHA2 XII-1:: Ecm33* | ST10575 | pCfB10314 | This study |
| ST10578 | *Δpdc5, Δaro10, ↑FjTAL, ↑EcAroL, ΔGPP1, ↑Aro4*, ↑Aro7*, ↑Aro1, ↑Aro2, ↑Aro3, ↑PHA2 XII-1:: Pun1* | ST10575 | pCfB10315 | This study |
| ST10579 | *Δpdc5, Δaro10, ↑FjTAL, ↑EcAroL, ΔGPP1, ↑Aro4*, ↑Aro7*, ↑Aro1, ↑Aro2, ↑Aro3, ↑PHA2 XII-1:: Kre2* | ST10575 | pCfB10316 | This study |
| ST10580 | *Δpdc5, Δaro10, ↑FjTAL, ↑EcAroL, ΔGPP1, ↑Aro4*, ↑Aro7*, ↑Aro1, ↑Aro2, ↑Aro3, ↑PHA2 XII-1:: Cwp1* | ST10575 | pCfB10317 | This study |
| ST10581 | *Δpdc5, Δaro10, ↑FjTAL, ↑EcAroL, ΔGPP1, ↑Aro4*, ↑Aro7*, ↑Aro1, ↑Aro2, ↑Aro3, ↑PHA2 XII-1:: Cwp2* | ST10575 | pCfB10318 | This study |
| ST10582 | *Δpdc5, Δaro10, ↑FjTAL, ↑EcAroL, ΔGPP1, ↑Aro4*, ↑Aro7*, ↑Aro1, ↑Aro2, ↑Aro3, ↑PHA2 XII-1:: Las21* | ST10575 | pCfB10319 | This study |
| ST10583 | *Δpdc5, Δaro10, ↑FjTAL, ↑EcAroL, ΔGPP1, ↑Aro4*, ↑Aro7*, ↑Aro1, ↑Aro2, ↑Aro3, ↑PHA2 XII-1:: Ecm33* | ST10575 | pCfB10320 | This study |
| ST10584 | *Δpdc5, Δaro10, ↑FjTAL, ↑EcAroL, ΔGPP1, ↑Aro4*, ↑Aro7*, ↑Aro1, ↑Aro2, ↑Aro3, ↑PHA2 XII-1:: Pun1* | ST10575 | pCfB10321 | This study |
| ST10585 | *Δpdc5, Δaro10, ↑FjTAL, ↑EcAroL, ΔGPP1, ↑Aro4*, ↑Aro7*, ↑Aro1, ↑Aro2, ↑Aro3, ↑PHA2 XII-1:: Kre2* | ST10575 | pCfB10322 | This study |
| ST10586 | *Δpdc5, Δaro10, ↑FjTAL, ↑EcAroL, ΔGPP1, ↑Aro4*, ↑Aro7*, ↑Aro1, ↑Aro2, ↑Aro3, ↑PHA2 XII-1:: Cwp1* | ST10575 | pCfB10323 | This study |
| ST10587 | *Δpdc5, Δaro10, ↑FjTAL, ↑EcAroL, ΔGPP1, ↑Aro4*, ↑Aro7*, ↑Aro1, ↑Aro2, ↑Aro3, ↑PHA2 XII-1:: Cwp2* | ST10575 | pCfB10324 | This study |
|  |  |  |  |  |

Table S2: Plasmids used in this study

| **Plasmid** | **Description** | **Parental vector** | **BioBricks** | **Source** |
| --- | --- | --- | --- | --- |
| pCfB2197 | pXII-1-loxP-NatMXsyn |  |  | (Stovicek et al., 2015) |
| pCfB10313 | IntXII-1_PrLas21->Las21 | pCfB2197 | BB4908, BB4909 | This study |
| pCfB10314 | IntXII-1_PrEcm33->Ecm33 | pCfB2197 | BB4910, BB4911 | This study |
| pCfB10315 | IntXII-1_PrPun1->Pun1 | pCfB2197 | BB4912, BB4913 | This study |
| pCfB10316 | IntXII-1_PrKre2->Kre2 | pCfB2197 | BB4914 ,BB4915 | This study |
| pCfB10317 | IntXII-1_PrCwp1->Cwp1 | pCfB2197 | BB4916, BB4917 | This study |
| pCfB10318 | IntXII-1_PrCwp2->Cwp2 | pCfB2197 | BB4918, BB4919 | This study |
| pCfB10319 | IntXII-1_PrTEF1->Las21 | pCfB2197 | B4920, BB4909 | This study |
| pCfB10320 | IntXII-1_PrTEF1->Ecm33 | pCfB2197 | BB4920, BB4911 | This study |
| pCfB10321 | IntXII-1_PrTEF1->Pun1 | pCfB2197 | BB4920, BB4913 | This study |
| pCfB10322 | IntXII-1_PrTEF1->Kre2 | pCfB2197 | BB4920, BB4915 | This study |
| pCfB10323 | IntXII-1_PrTEF1->Cwp1 | pCfB2197 | BB4920, BB4917 | This study |
| pCfB10324 | IntXII-1_PrTEF1->Cwp2 | pCfB2197 | BB4920, BB4919 | This study |
| P0027 | TEF1, GPK1 promoters |  |  | (Partow et al., 2010) |
|  |  |  |  |  |

Table S3: Biobricks used in this study

| **BioBrick** | **Description** | **PCR template** | **Forward primer** | **Reverse primer** |
| --- | --- | --- | --- | --- |
| BB4526 | KANMX4 with las21 homology regions | gDNA 1361 | PR-25422 (Las21F1) | PR-25425 (Las21R2) |
| BB4527 | KANMX4 with ecm33 homology regions | gDNA 3215 | PR-25426 (Ecm33F1) | PR-25429 (Ecm33R2) |
| BB4528 | KANMX4 with pun1 homology regions | gDNA 5322 | PR-25430 (Pun1F1) | PR-25433 (Pun1R2) |
| BB4798 | KANMX4 with cwp1 homology regions | gDNA 4945 | PR-27244 (Cwp1F1) | PR-27245 (Cwp1R1) |
| BB4799 | KANMX4 with cwp2 homology regions | gDNA 7026 | PR-27246 (Cwp2F1) | PR-27247 (Cwp2R1) |
| BB4800 | KANMX4 with kre2 homology regions | gDNA 4317 | PR-27242 (Kre2F1) | PR-27243 (Kre2R1) |
| BB4908 | Las21 native promoter | gDNA ST9769 | PR-27671 (PLas21Fw) | PR-27672 (PLas21Rv) |
| BB4909 | Las21 | gDNA ST9769 | PR-27673 (CS_Las21Fw) | PR-27674 (CS_Las21Rv) |
| BB4910 | Ecm33 native promoter | gDNA ST9769 | PR-27675 (PEcm33Fw) | PR-27676 (PEcm33Rv) |
| BB4911 | Ecm33 | gDNA ST9769 | PR-27677 (CS_Ecm33Fw) | PR-27678(CS_Ecm33Rv) |
| BB4912 | Pun1 native promoter | gDNA ST9769 | PR-27679 (PPun1Fw) | PR-27680 (PPun1Rv) |
| BB4913 | Pun1 | gDNA ST9769 | PR-27681(CS_Pun11Fw) | PR-27682 (CS_Pun1Rv) |
| BB4914 | Kre2 native promoter | gDNA ST9769 | PR-27683(PKre2Fw) | PR-27684 (PKre2Rv) |
| BB4915 | Kre2 | gDNA ST9769 | PR-27685(CS_Kre2Fw) | PR-27686(CS_Kre2Rv) |
| BB4916 | Cwp1 native promoter | gDNA ST9769 | PR-27687(PCwp1Fw) | PR-27688 (PCwp1Rv) |
| BB4917 | Cwp1 | gDNA ST9769 | PR-27689 (CS_Cwp1Fw) | PR-27690(CS_Cwp1Rv) |
| BB4918 | Cwp2 native promoter | gDNA ST9769 | PR-27691(PCwp2Fw) | PR-27692(PCwp2Rv) |
| BB4919 | Cwp2 | gDNA ST9769 | PR-27693 (CS_Cwp2Fw) | PR-27694 (CS_Cwp2Rv) |
| BB4920 | TEF1 promoter | p0027 | PR-22408(pTEF_P2_Fwd) | PR-22409(pTEF_P2_Rev) |
|  |  |  |  |  |

Table S4: Primers used in this study

| **Primer** | **Sequence** | **Description** |
| --- | --- | --- |
| PR-25422 (Las21F1) | GCTGTTCTTCTCTCCTCTTTTGTAA | Forward primer for amplifying KanMX4 from *Δlas21* YKO Collection |
| PR-25425 (Las21R2) | GCACTTAACCTACTAGATTGGGACA | R primer for amplifying KanMX from *Δlas21* YKO Collection |
| PR-25426 (Ecm33F1) | AGCCGGTATAAATATTCAATGTCAA | Forward primer for amplifying KanMX4 from *Δecm33* YKO Collection |
| PR-25429 (Ecm33R2) | AGAAGAGCAGTAAAGATGGCAGTTA | Reverse primer for amplifying KanMX4 from *Δecm33* YKO Collection |
| PR-25430 (Pun1F1) | TATTTCCAAATCGGGCGTACTAT | Reverse primer for amplifying KanMX4 from *Δpun1* YKO Collection |
| PR-25433 (Pun1R2) | ATTATTTCTGGCTCTTCTCCATTTT | Reverse primer for amplifying KanMX4 from *Δpun1* YKO Collection |
| PR-26424 (Las21F1.2) | TTAACCAACTTTTCCCGCCT | Forward primer to check las21 deletion |
| PR-26425 (Ecm33F1.2) | TTCTTTTTCTTCGTCTCTCTCTCC | Forward primer to check ecm33 deletion |
| PR-26426 (Pun1F1.2) | GCGAACGTCACAGATCAAAA | Forward primer to check *pun1* deletion |
| PR-26827 (Las21R1.2) | TGTAACGTGGAGATTGGCTTG | Reverse primer to check *las21* deletion |
| PR-26828 (Ecm33R1.2) | CAACCCCAACTCCATCTTCT | Reverse primer to check *ecm33* deletion |
| PR-26829 (Pun1R1.2) | TCCGTGGTGAGTCAAAAATTA | Reverse primer to check *pun1* deletion |
| PR-27242 (Kre2F1) | TTATAGTGCCATTCTTGAGGTC | Forward primer for amplifying KanMX4 from *ΔKre2* YKO Collection |
| PR-27243 (Kre2R1) | TTCCTCTTTCCTCTCACTTGT | Reverse primer for amplifying KanMX4 from *Δkre2* YKO Collection |
| PR-27244 (Cwp1F1) | GAAGGTGCCACAAAAGAAAAC | Forward primer for amplifying KanMX4 from *Δcwp1* YKO Collection |
| PR-27245 (Cwp1R1) | CGTATTAGCAGTCAGTGGAAC | Reverse primer for amplifying KanMX4 from *Δcwp1* YKO Collection |
| PR-27246 (Cwp2F1) | CCGTCCTTTTTCCTCTTTCT | Forward primer for amplifying KanMX4 from *Δcwp2* YKO Collection |
| PR-27247 (Cwp2R1) | AGCAAACTCGAAGAAACCCA | Reverse primer for amplifying KanMX4 from *Δcwp2* YKO Collection |
| PR-27398 (Kre2F2) | CTTGAGCCCTATTCCGTTGTA | Forward primer to check *kre2* deletion |
| PR-27399 (Kre2R2) | ACACCTCTTTGAGAACATCC | Reverse primer to check *kre2* deletion |
| PR-27400 (Cwp1F2) | TCCCACAACGGTAACTTACT | Forward primer to check *cwp1* deletion |
| PR-27401 (Cwp1R2) | ACCGTAGTGATTTCTTGCTT | Reverse primer to check *cwp1* deletion |
| PR-27402 (Cwp2F2) | AACATCCCACTACCCTAGAAA | Forward primer to check *cwp2* deletion |
| PR-27403 (Cwp2R2) | CACGCCTTGACTCTTTAATC | Reverse primer to check cwp2 deletion |
| PR-27671 (PLas21Fw) | CGTGCGAUTAATGATCCGCTCTGCGACCCT | Forward primer to amplify las21 native promoter |
| PR-27672 (PLas21Rv) | ATGACAGAUCTTGCTAACTTGGTGAAGTGTGCG | Reverse primer to amplify las21 native promoter |
| PR-27673 (CS_Las21Fw) | ATCTGTCAUAAAACAATGAACTTGAAGCAGTTCACGTGCC | Forward primer to amplify las21 |
| PR-27674 (CS_Las21Rv) | CACGCGAUTTAATCAAGAGCGCAAAGGAGGGC | Reverse primer to amplify las21 |
| PR-27675 (PEcm33Fw) | CGTGCGAUGCATAGGACAGTATACCCCCA | Forward primer to amplify ecm33 native promoter |
| PR-27676 (PEcm33Rv) | ATGACAGAUTGCGGAATAATAGTAAAATTAAAACTAGATCTTTA | Reverse primer to amplify ecm33 native promoter |
| PR-27677 (CS_Ecm33Fw) | TCTGTCAUAAAACAATGCAATTCAAGAACGCTTTGACTGCT | Forward primer to amplify ecm33 |
| PR-27678(CS_Ecm33Rv) | CACGCGAUTTATAGTAAGGCAACGCCAACAGCAG | Reverse primer to amplify ecm33 |
| PR-27679 (PPun1Fw) | CGTGCGAUGCCCCTCTGTCGCTGTAGAATCT | Forward primer to amplify pun1 native promoter |
| PR-27680 (PPun1Rv) | ATGACAGAUGCTTATAGCGTCCTTCGATGATGTGTT | Reverse primer to amplify pun1native promoter |
| PR-27681(CS_Pun11Fw) | ATCTGTCAUAAAACAATGAGGAATTTTTTCACGTTATTTTTTGCAGCTAT | Forward primer to amplify pun1 |
| PR-27682 (CS_Pun1Rv) | CACGCGAUTCAAATCAATGGTTTTTCCTCAATTGGAGC | Reverse primer to amplify pun1 |
| PR-27683(PKre2Fw) | CGTGCGAUCGTCTCTTTTTAGGACGAGAGCTCG | Forward primer to amplify kre2 native promoter |
| PR-27684 (PKre2Rv) | ATGACAGAUGCTTTCGAGTGGAACTGCTTGCG | Reverse primer to amplify kre2 native promoter |
| PR-27685(CS_Kre2Fw) | ATCTGTCAUAAAACAATGGCCCTCTTTCTCAGTAAGAGACTG | Forward primer to amplify kre2 |
| PR-27686(CS_Kre2Rv) | CACGCGAUCTACTCACGGAATTTTTTCCAGTTTTTTGGC | Reverse primer to amplify kre2 |
| PR-27687(PCwp1Fw) | CGTGCGAUTCCTCACTACAATTGCCCCTCGG | Forward primer to amplify cwp1 native promoter |
| PR-27688 (PCwp1Rv) | ATGACAGAUTATTGTTTTTTGAGACTTTCGTAGTATTTATTAGTTTGTTACGG | Reverse primer to amplify cwp1 native promoter |
| PR-27689 (CS_Cwp1Fw) | ATCTGTCAUAAAACAATGAAATTCTCCACTGCTTTGTCTGTCG | Forward primer to amplify cwp1 |
| PR-27690(CS_Cwp1Rv) | CACGCGAUTTACAACAAGTAAGCAGCTGCGACC | Reverse primer to amplify cwp1 |
| PR-27691(PCwp2Fw) | CGTGCGAUATGCTGCTCGTCTTTTTTTGACGACC | Forward primer to amplify cwp2 native promoter |
| PR-27692(PCwp2Rv) | ATGACAGAUTTTTTTTCTTGTTAGTGTGTAGCGAATGTGATG | Reverse primer to amplify cwp2 native promoter |
| PR-27693 (CS_Cwp2Fw) | ATCTGTCAUAAAACAATGCAATTCTCTACTGTCGCTTCCG | Forward primer to amplify cwp2 |
| PR-27694 (CS_Cwp2Rv) | CACGCGAUTTATAACAACATAGCAGCAGCAGCTAGAG | Reverse primer to amplify cwp2 |
| 891 | CTGGCAAGAGAACCACCAAT | Primer for integration site XII-1 |
| 892 | GGACGACAACTACGGAGGAT | Primer for integration site XII-1 |
| 2221 | GTTGACACTTCTAAATAAGCGAATTTC | Primer for integration site XII-1 |
| PR0044 | CACGGATAGTGGCTTTGGTGAACAATTAC | Fw ALG9 |
| PR0045 | TATGATTATCTGGCAGCAGGAAAGAACTTGGG | RV ALG9 |
| PR0120 | CATTTTGACCAGTGCTTCTTTCGGTTCTTT | Fw primer ecm33 qPCR |
| PR0121 | GATATCTGTTGTTGTTGATGTTAAAAACATTAACCTTCTTCA | Rv primer ecm33 qPCR |
|  |  |  |

**Supplementary information 2**


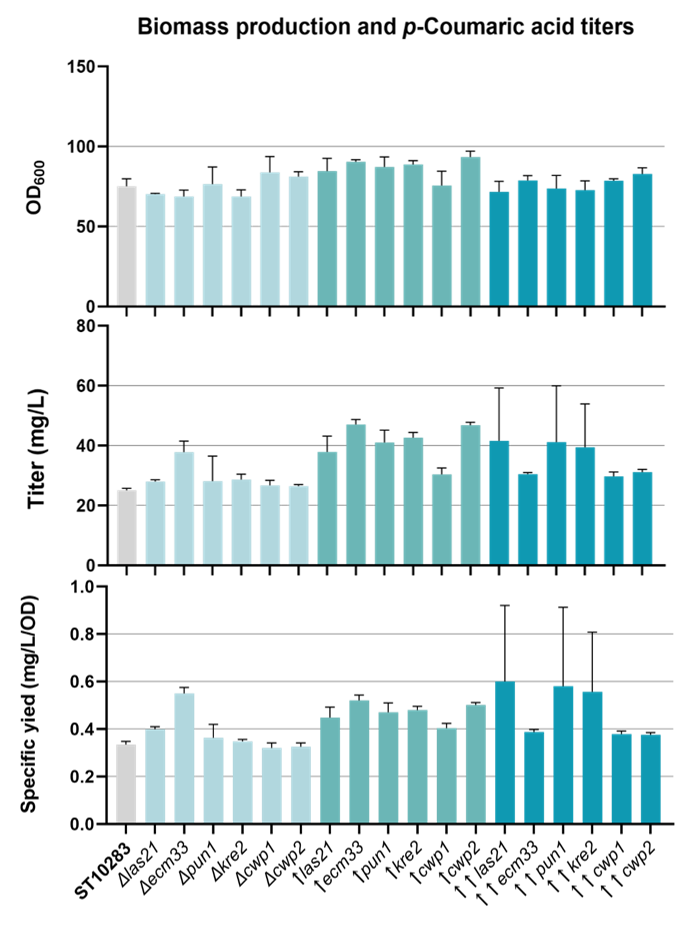


**Fig S1. Biomass production, titers and specific yield of *p*-coumaric acid in cell wall engineered *S. cerevisiae* strains.** Cultivations were carried out for 72 h in 24 deep-well plates containing Feed-In-Time medium with 60 g/L of polysaccharide. Extracellular content was subjected to HPLC analysis. Error bars represent standard deviation from three biological replicates. The symbol ↑ represents upregulation with a double copy integration with the native promoter and ↑↑ represents *TEF1* promoter.


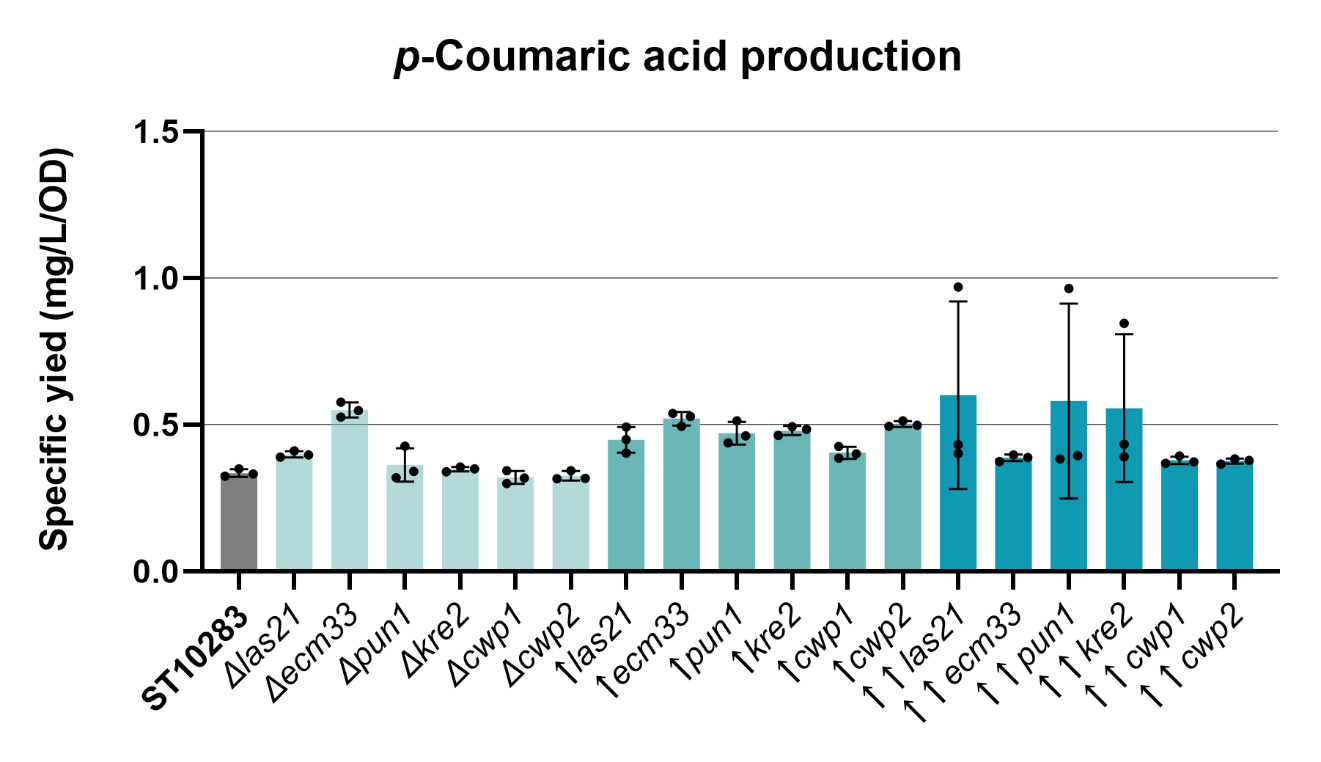


**Fig S1.1. Specific yield of *p*-coumaric acid in cell wall engineered *S. cerevisiae* strains with individual data points.** Cultivations were carried out for 72 h in 24 deep-well plates containing Feed-In-Time medium with 60 g/L of polysaccharide. Extracellular content was subjected to HPLC analysis. Error bars represent standard deviation from three biological replicates. The symbol ↑ represents upregulation with a double copy integration with the native promoter and ↑↑ represents *TEF1* promoter.


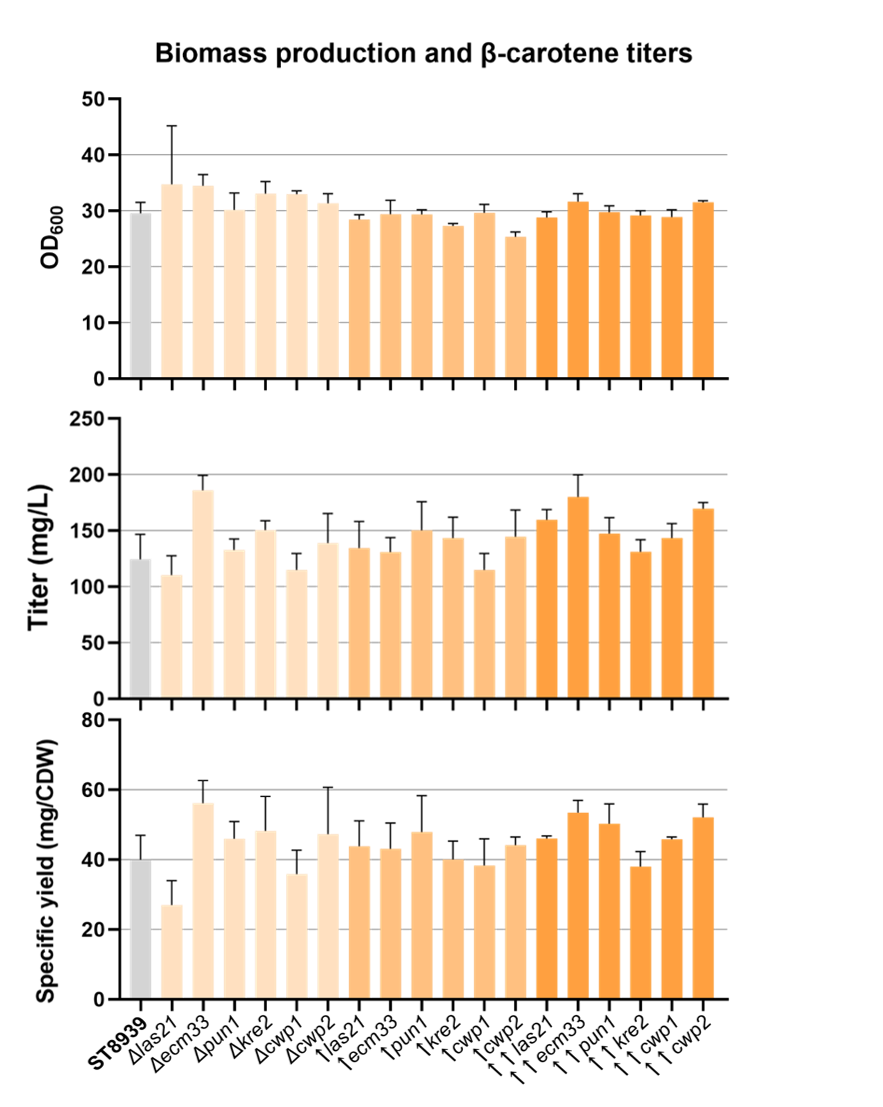


**Fig S2. Biomass production, titers and specific yield of β-carotene in cell wall engineered *S. cerevisiae* strains.** Cultivations were carried out for 72 h in 24 deep-well plates containing YP 8 % D-glucose. Intracellular content was subjected to HPLC analysis. Error bars represent standard deviation from three biological replicates. The symbol ↑ represents upregulation with a double copy integration with the native promoter and ↑↑ represents *TEF1* promoter.


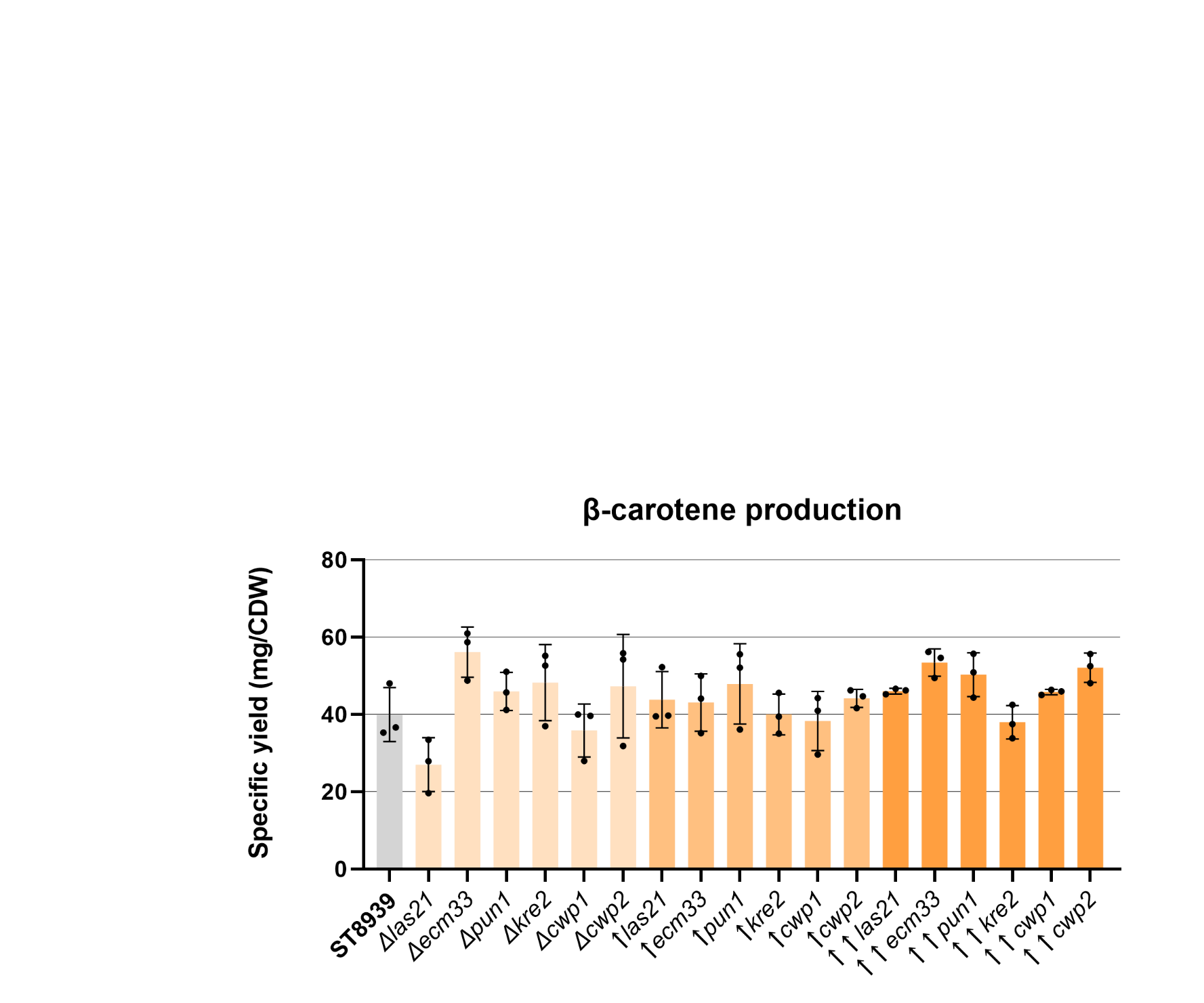


**Fig S2.1 Specific yield of β-carotene in cell wall engineered *S. cerevisiae* strains with individual data points.** Cultivations were carried out for 72 h in 24 deep-well plates containing YP 8 % D-glucose. Intracellular content was subjected to HPLC analysis. Error bars represent standard deviation from three biological replicates. The symbol ↑ represents upregulation with a double copy integration with the native promoter and ↑↑ represents *TEF1* promoter.

***p*-Coumaric acid intracellular titer**


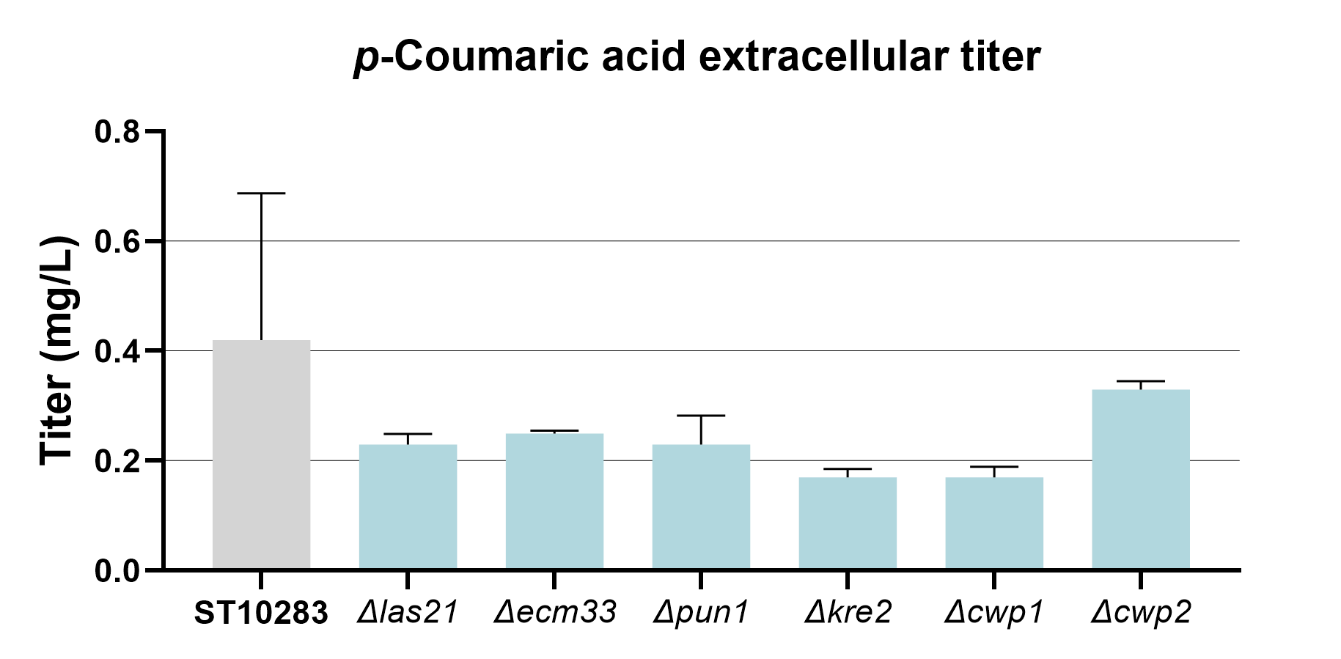


**Fig S3. Intracellular production of p-Coumaric acid in cell wall mutants.** Cultivations were carried out for 72 h in 24 deep-well plates containing Feed-In-Time medium with 60 g/L of polysaccharide. Intracellular content was subjected to HPLC analysis. Error bars represent standard deviation from three biological replicates.


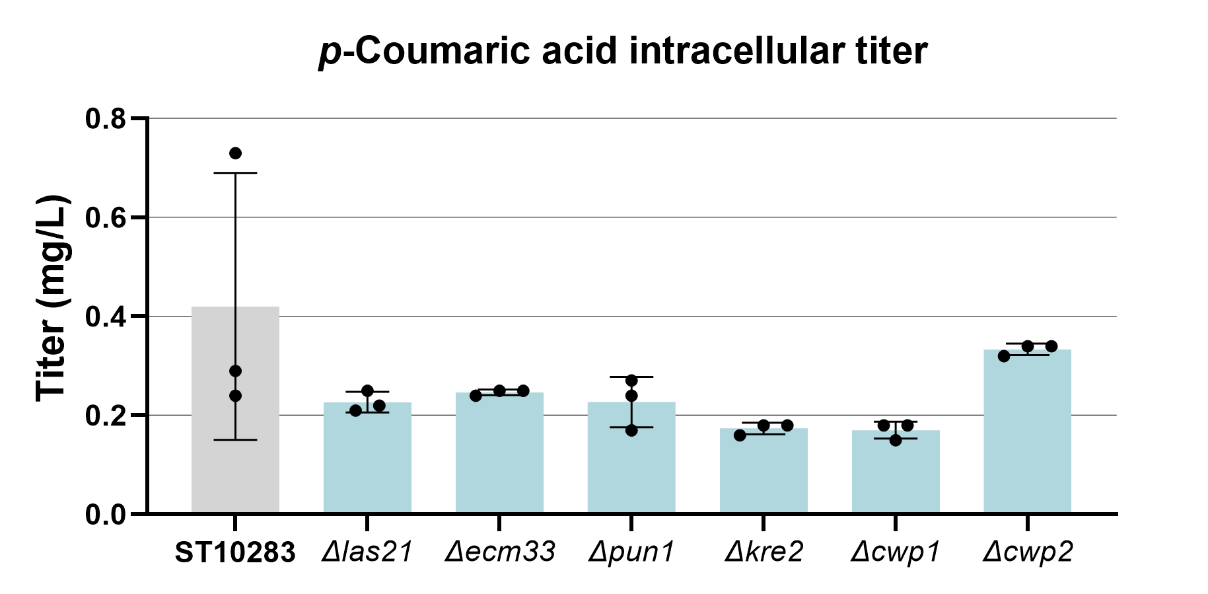


**Fig S3.1. Intracellular production of p-Coumaric acid in cell wall mutants with individual data points.** Cultivations were carried out for 72 h in 24 deep-well plates containing Feed-In-Time medium with 60 g/L of polysaccharide. Intracellular content was subjected to HPLC analysis. Error bars represent standard deviation from three biological replicates.


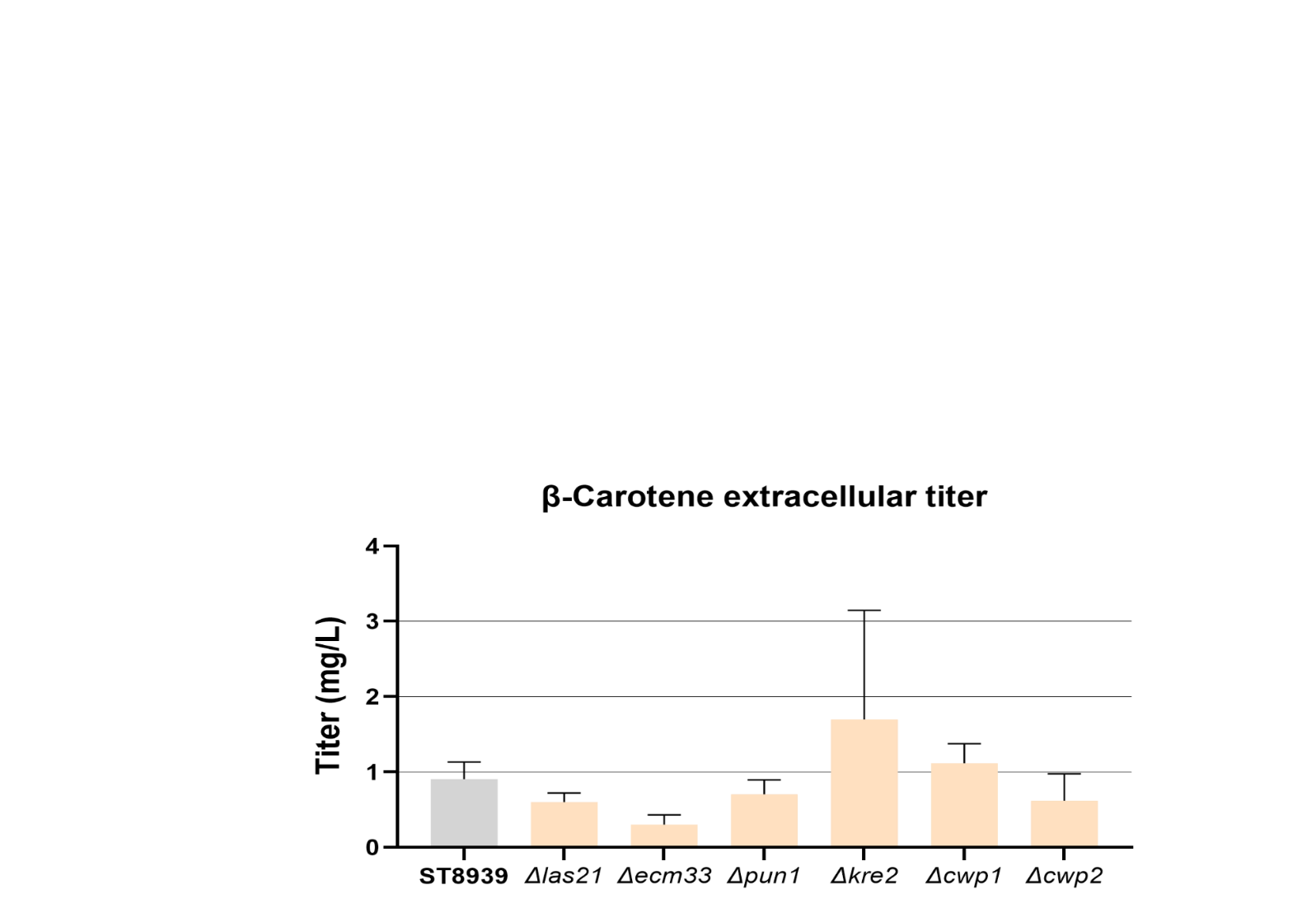


**Fig S4. Extracellular production of β-carotene in cell wall mutants.** Cultivations were carried out for 72 h in YP 8 % D-glucose and the supernatant was mixed with 100 μL of dodecane. . Extracellular content in the dodecane layer was subjected to HPLC analysis. Error bars represent standard deviation from three biological replicates.


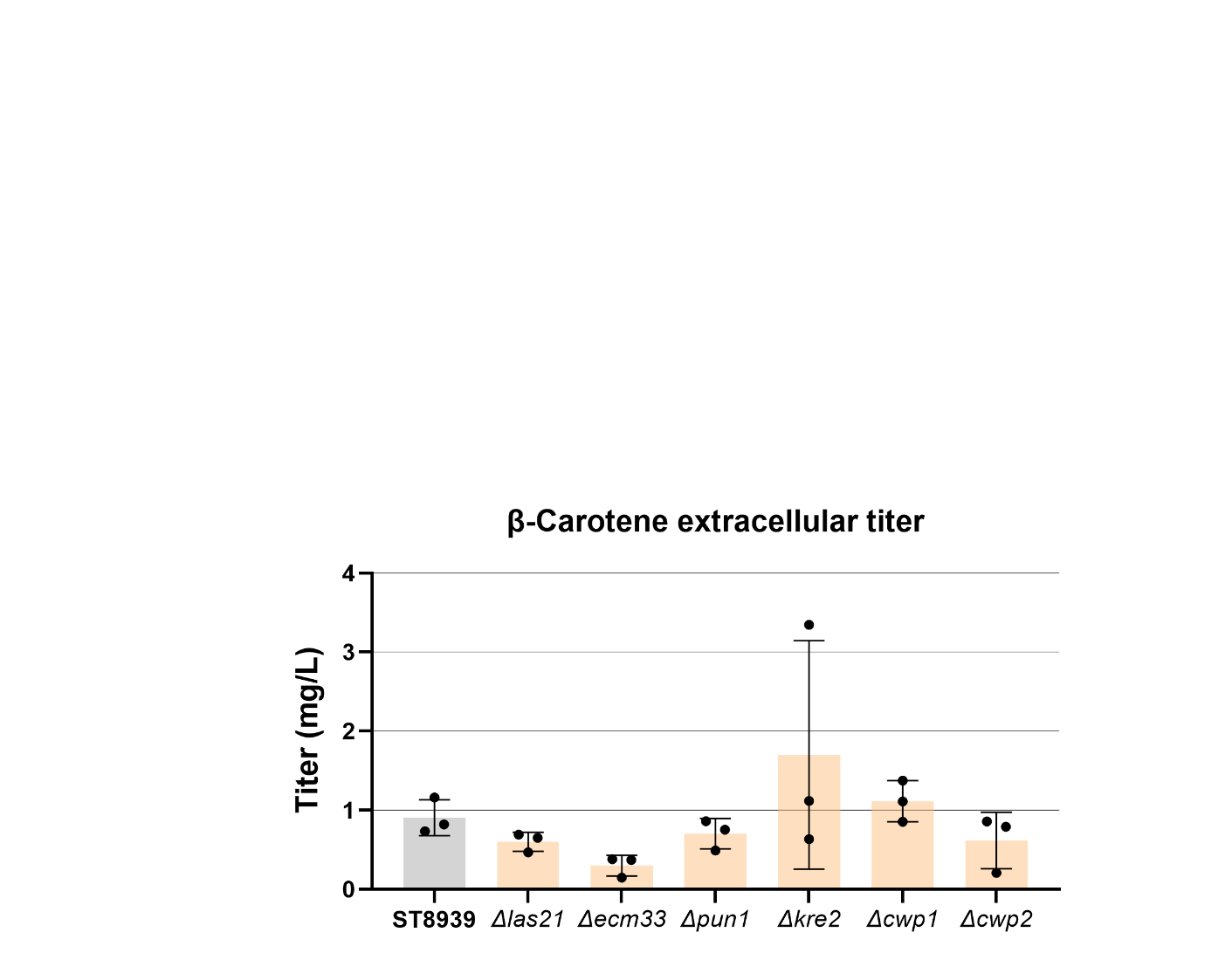


**Fig S4.1.** **Extracellular production of β-carotene in cell wall mutants with individual data points.** Cultivations were carried out for 72 h in YP 8 % D-glucose and the supernatant was mixed with 100 μL of dodecane. . Extracellular content in the dodecane layer was subjected to HPLC analysis. Error bars represent standard deviation from three biological replicates.


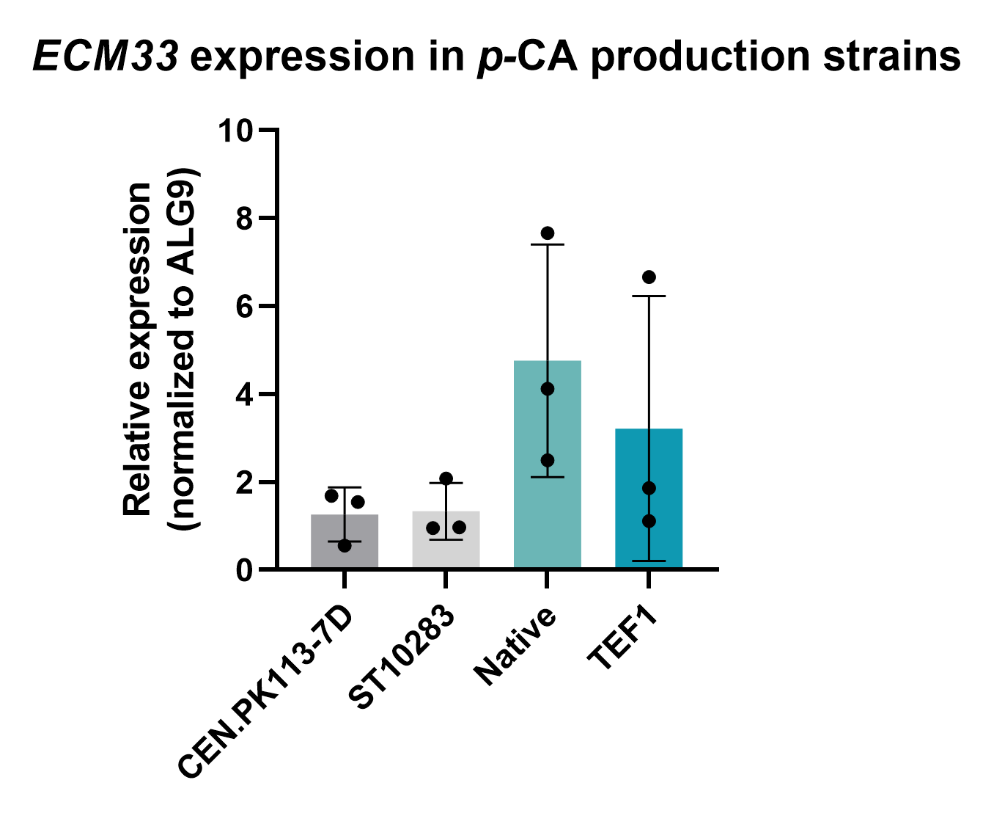


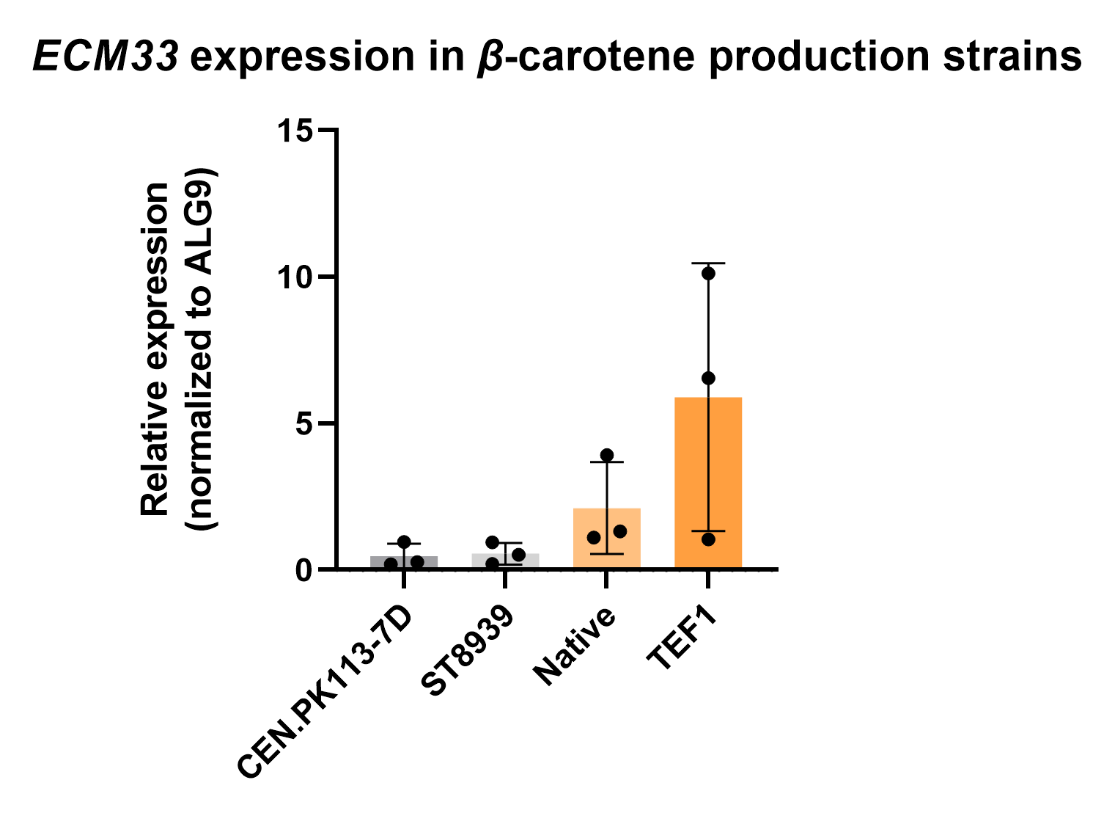


**Fig S5.** ***ECM33* expression in *p*-CA and β-carotene production strains with individual data points.** Expression of ECM33 was measured by qPCR after 16h of growth. The expression values are normalized to the house-keeping gene ALG9.

**References**

Entian, K.-D., & Kötter, P. (2007). 25 Yeast Genetic Strain and Plasmid Collections. *Methods in Microbiology*, *36*, 629–666. https://doi.org/10.1016/S0580-9517(06)36025-4

Milne, N., Tramontin, L. R. R., & Borodina, I. (2020). A teaching protocol demonstrating the use of EasyClone and CRISPR/Cas9 for metabolic engineering of Saccharomyces cerevisiae and Yarrowia lipolytica. *FEMS Yeast Research*, *20*(2). https://doi.org/10.1093/femsyr/foz062

Partow, S., Siewers, V., Bjørn, S., Nielsen, J., & Maury, J. (2010). Characterization of different promoters for designing a new expression vector in Saccharomyces cerevisiae. *Yeast (Chichester, England)*, *27*(11), 955–964. https://doi.org/10.1002/yea.1806

Stovicek, V., Borja, G. M., Forster, J., & Borodina, I. (2015). EasyClone 2.0: expanded toolkit of integrative vectors for stable gene expression in industrial Saccharomyces cerevisiae strains. *Journal of Industrial Microbiology & Biotechnology*, *42*(11), 1519–1531. https://doi.org/10.1007/s10295-015-1684-8
