# Supplementary figures and images for: Modulation of the cell wall protein Ecm33p in yeast Saccharomyces cerevisiae improves the production of small metabolites
Source: FEMS Yeast Res. 2022 Aug 3;22(1):foac037. doi: 10.1093/femsyr/foac037 (PMC9440718; doi:10.1093/femsyr/foac037)

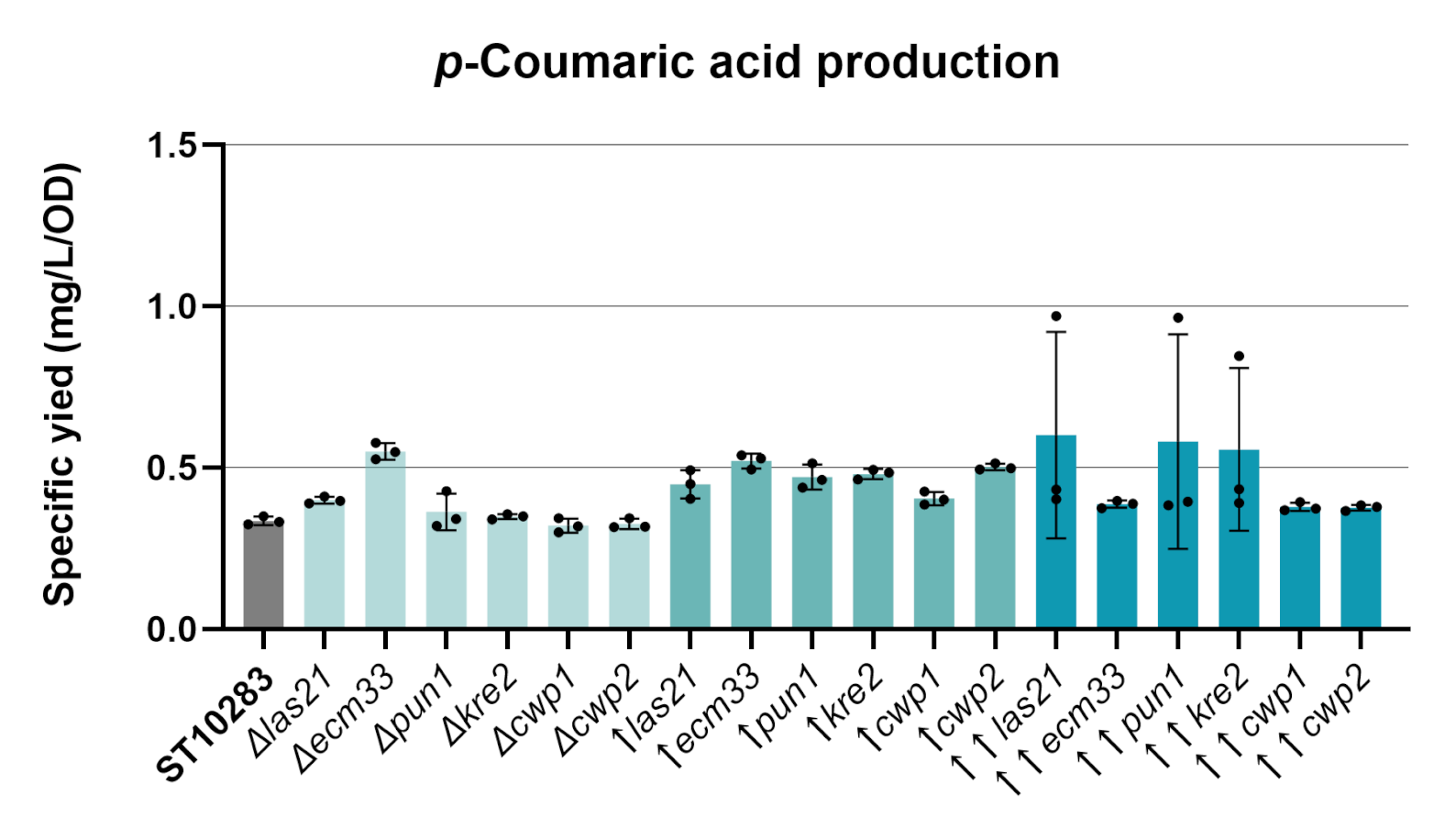

Supplement: foac037_Supplemental_files [file foac037_supplemental_files.zip › Figure_S1.1.tif]

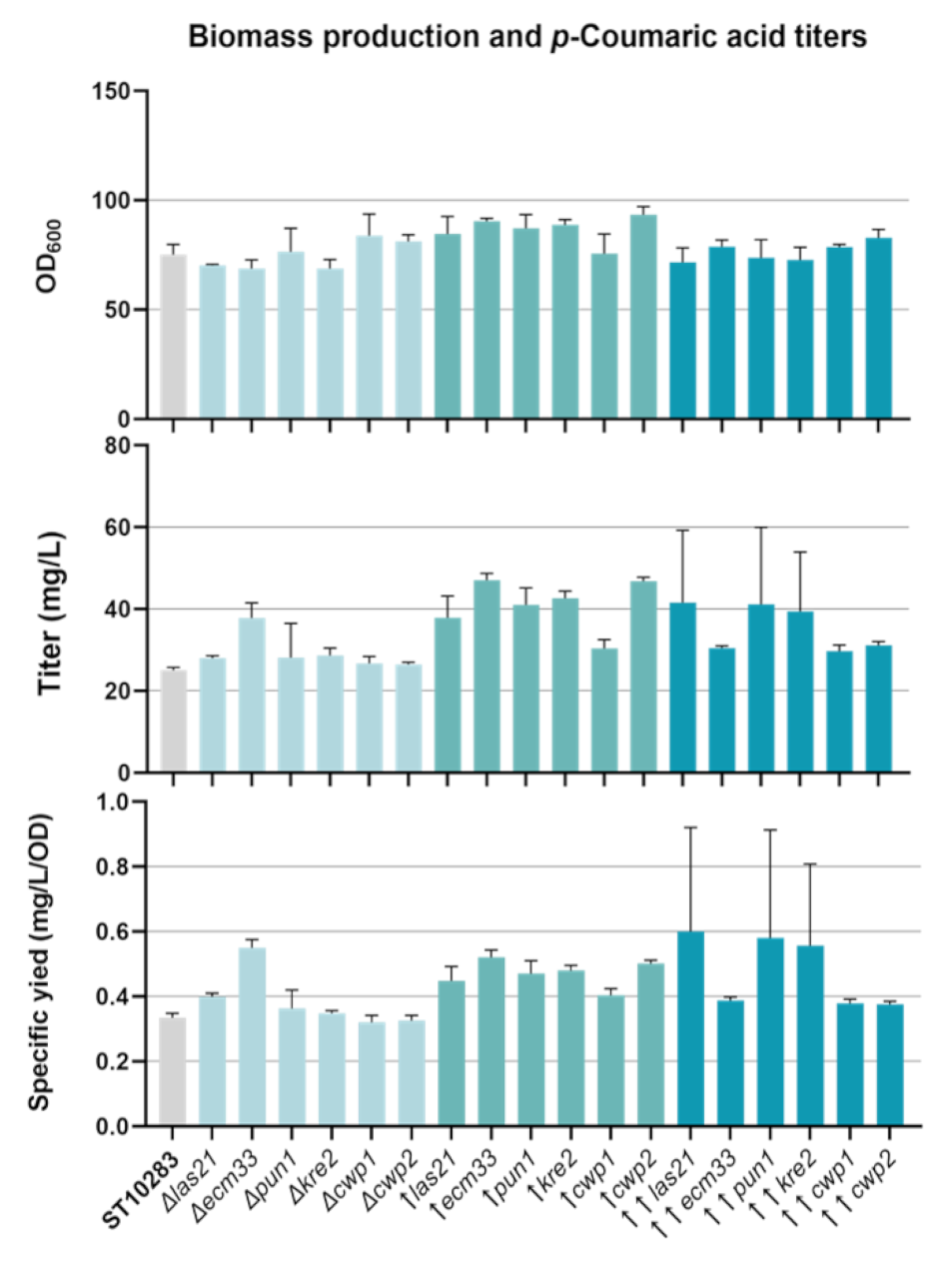

Supplement: foac037_Supplemental_files [file foac037_supplemental_files.zip › Figure_S1.tif]

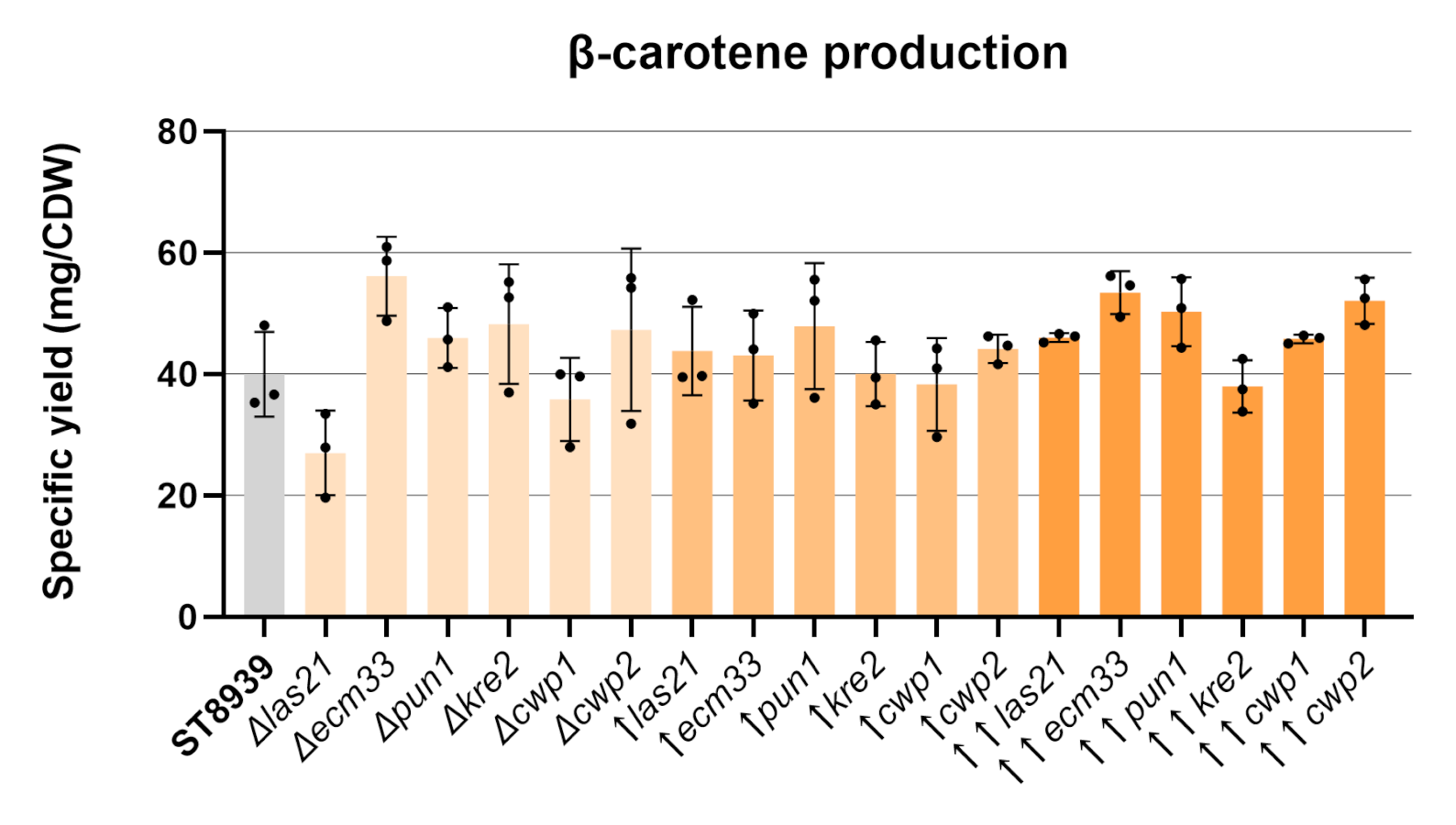

Supplement: foac037_Supplemental_files [file foac037_supplemental_files.zip › Figure_S2.1.tif]

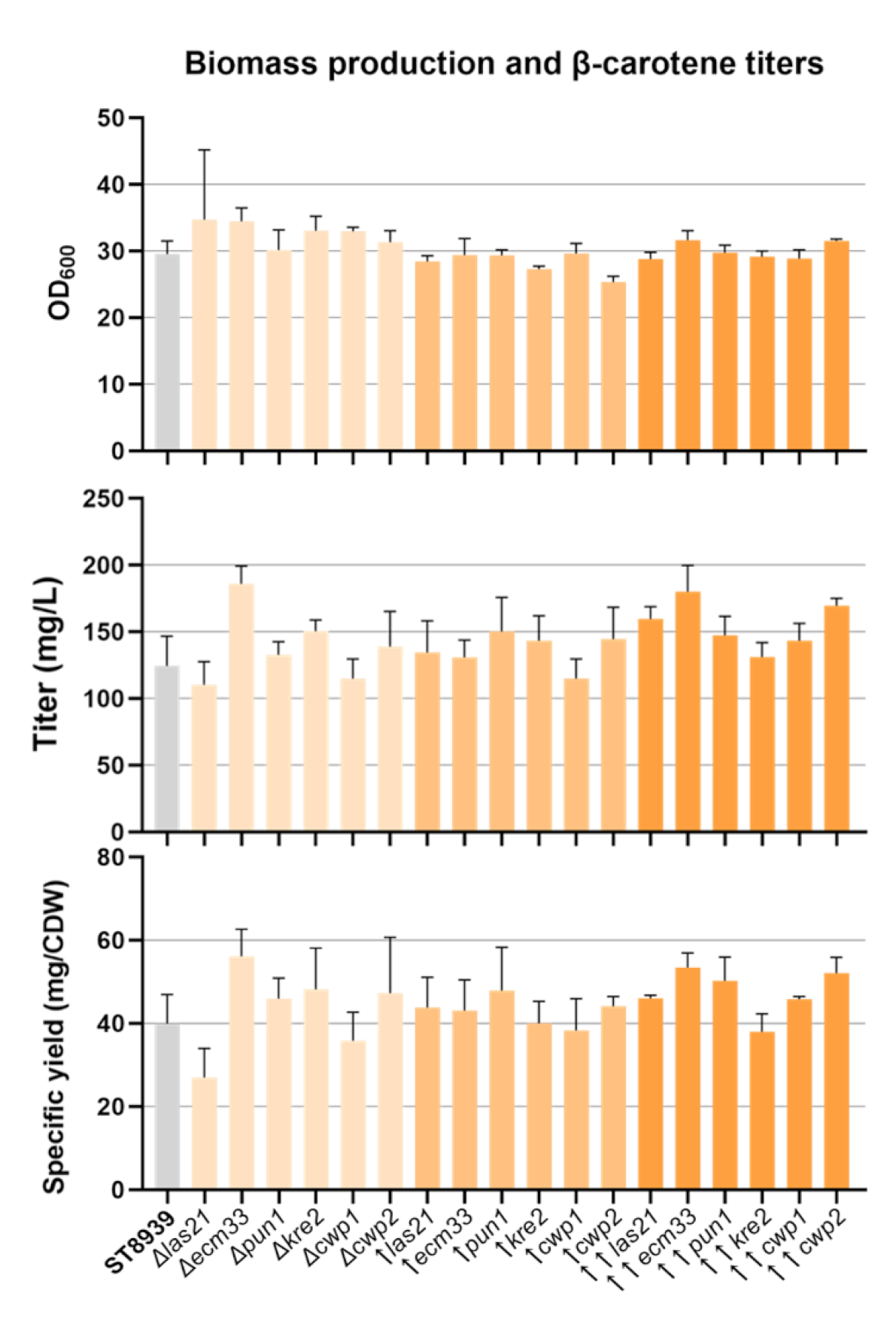

Supplement: foac037_Supplemental_files [file foac037_supplemental_files.zip › Figure_S2.tif]

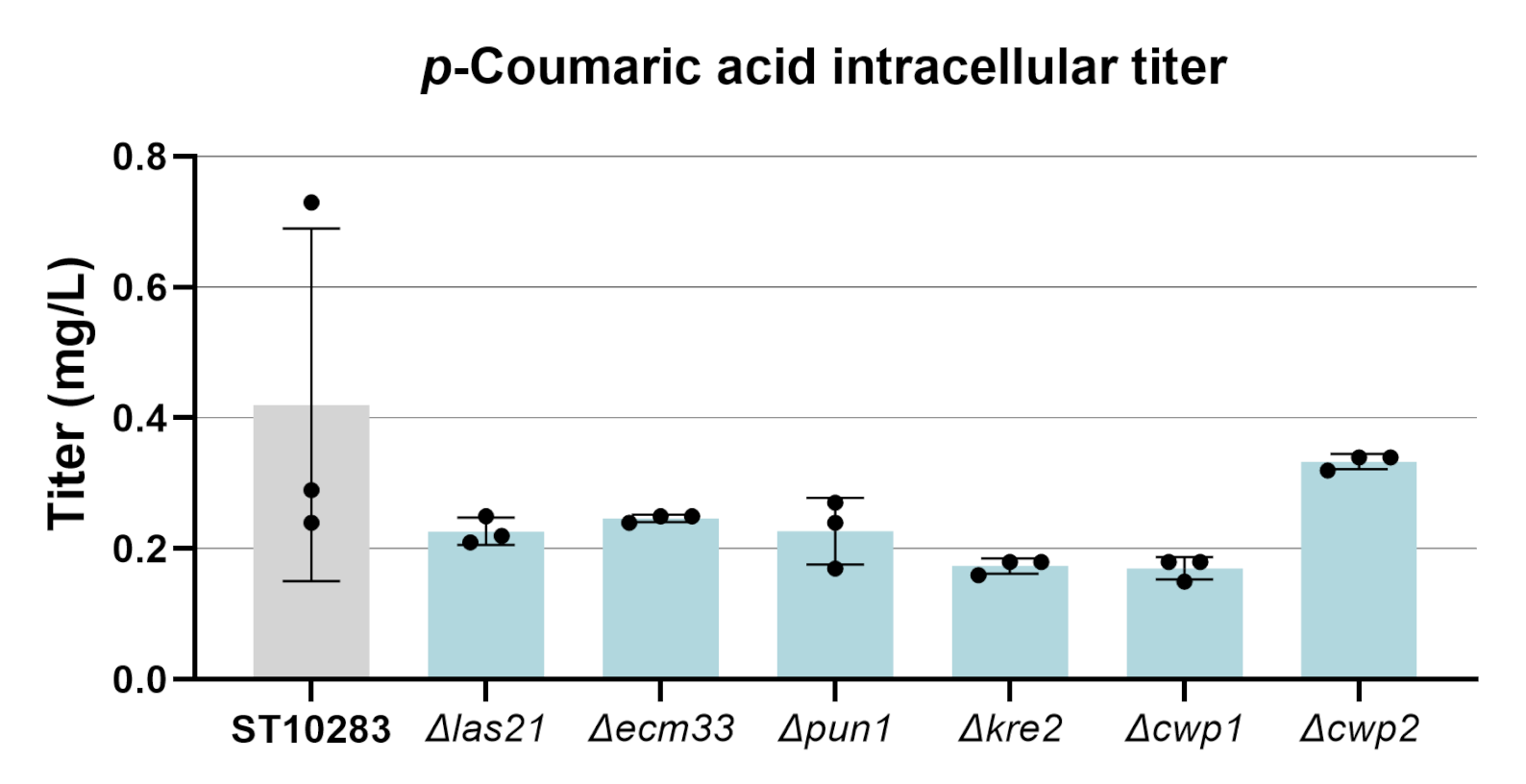

Supplement: foac037_Supplemental_files [file foac037_supplemental_files.zip › Figure_S3.1.tif]

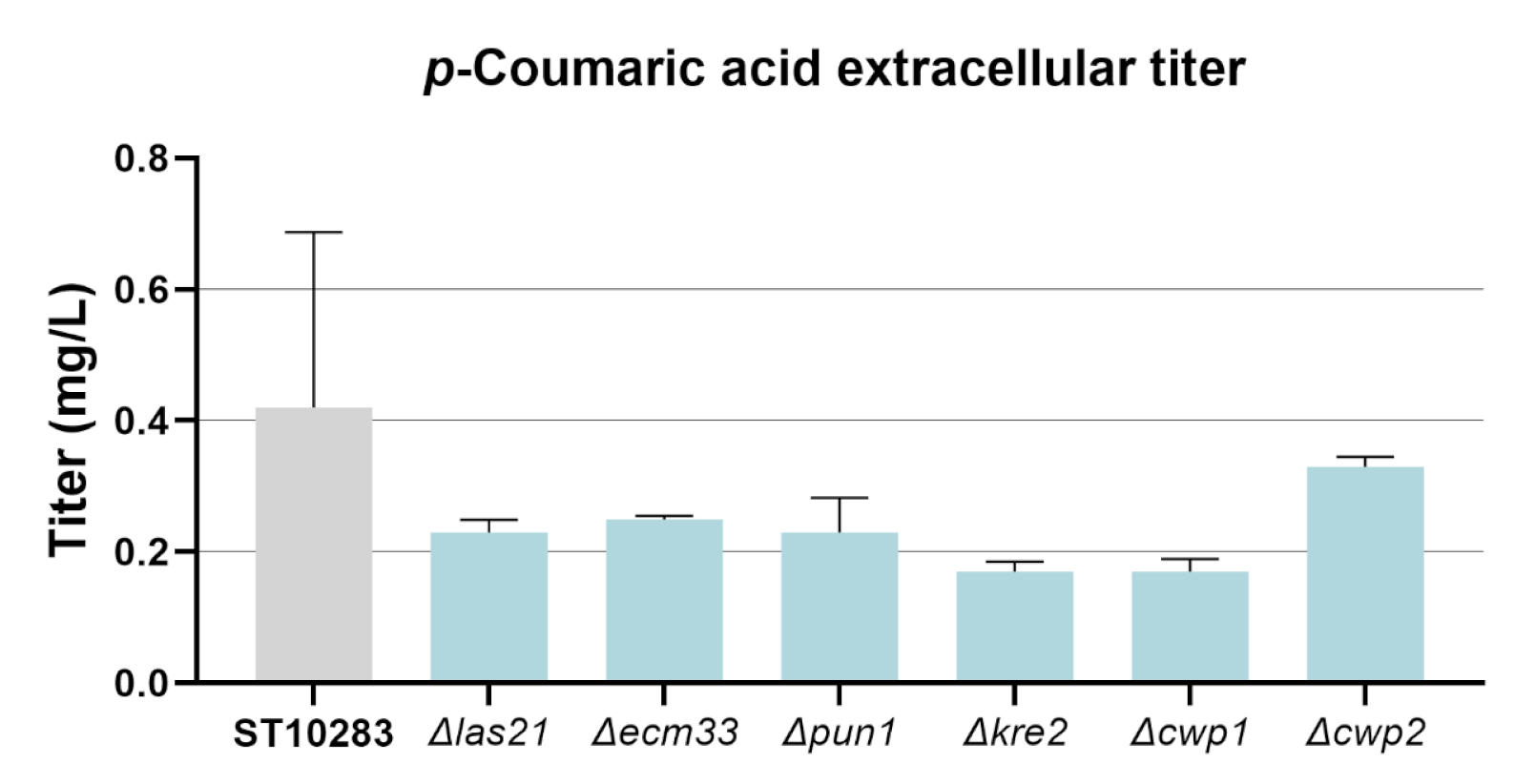

Supplement: foac037_Supplemental_files [file foac037_supplemental_files.zip › Figure_S3.tif]

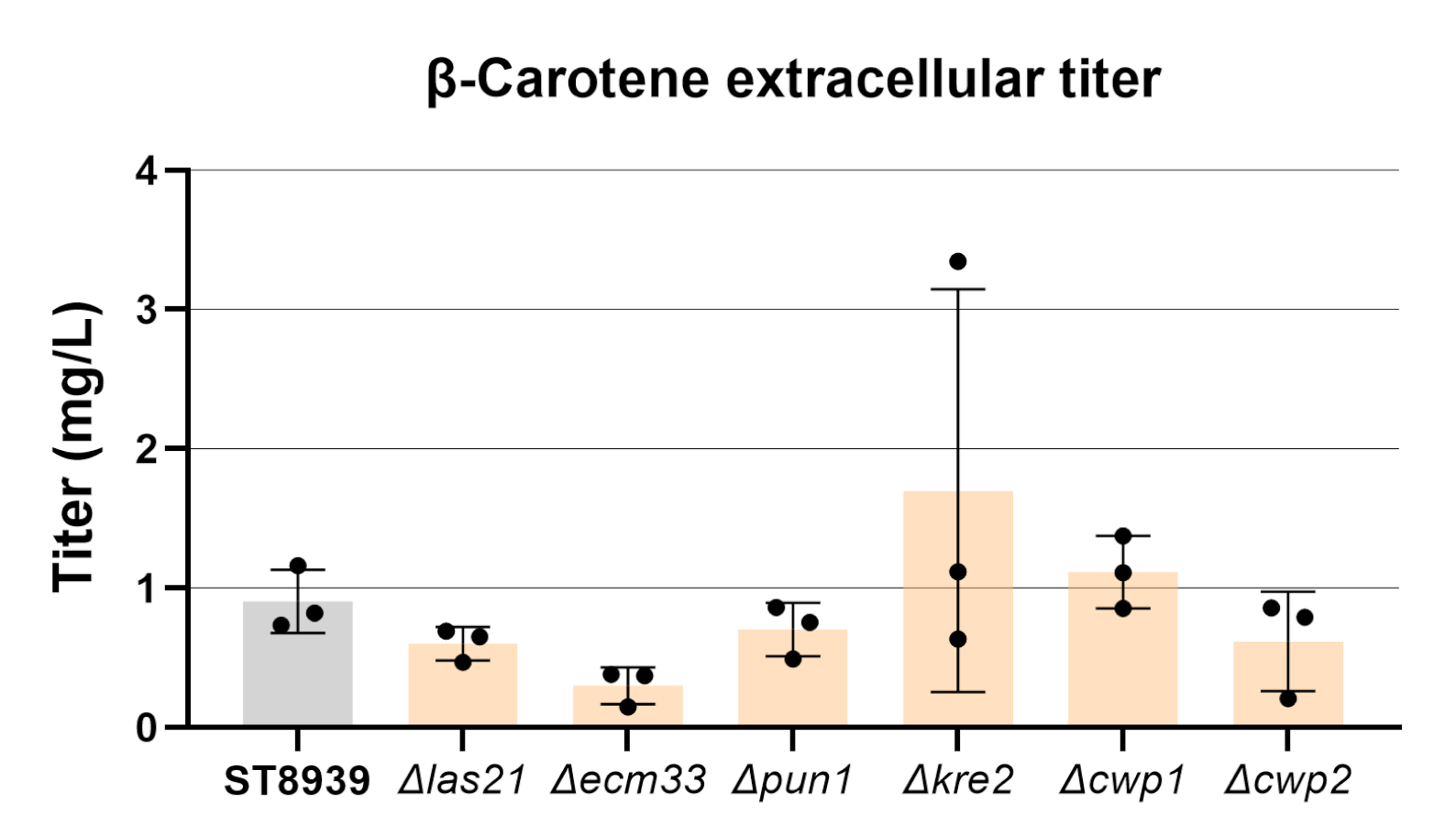

Supplement: foac037_Supplemental_files [file foac037_supplemental_files.zip › Figure_S4.1.tif]

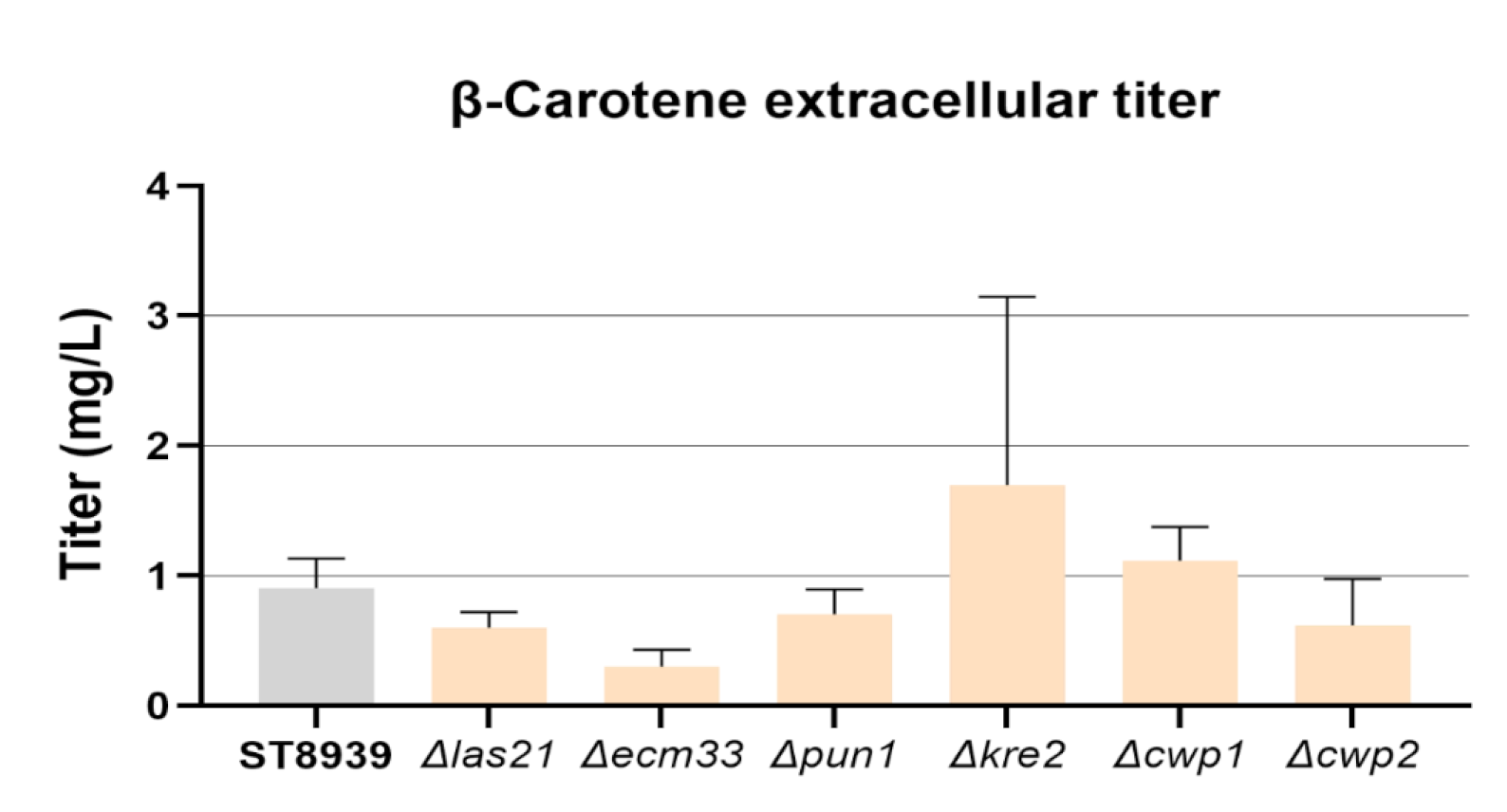

Supplement: foac037_Supplemental_files [file foac037_supplemental_files.zip › Figure_S4.tif]

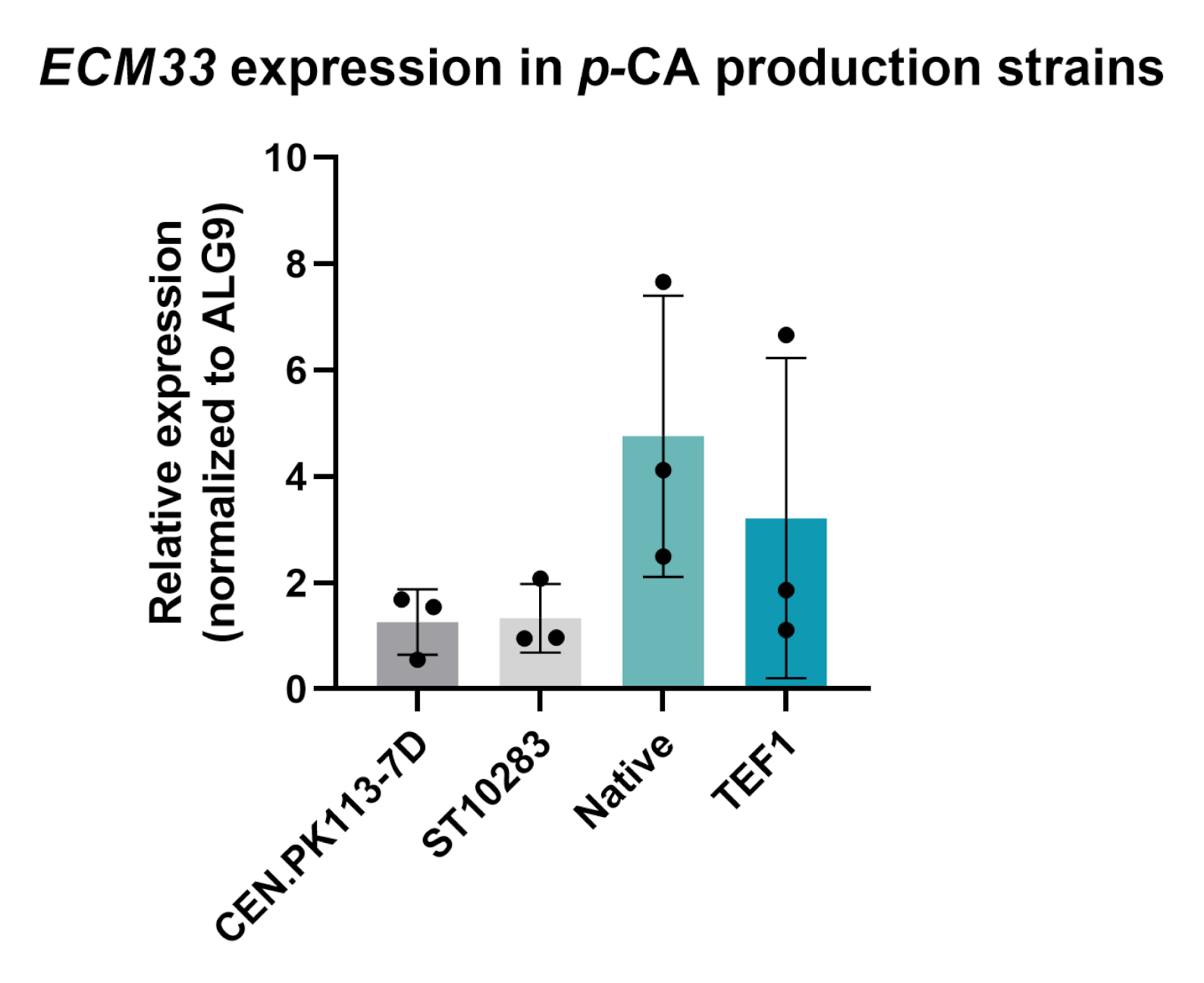

Supplement: foac037_Supplemental_files [file foac037_supplemental_files.zip › Figure_S5a.tif]

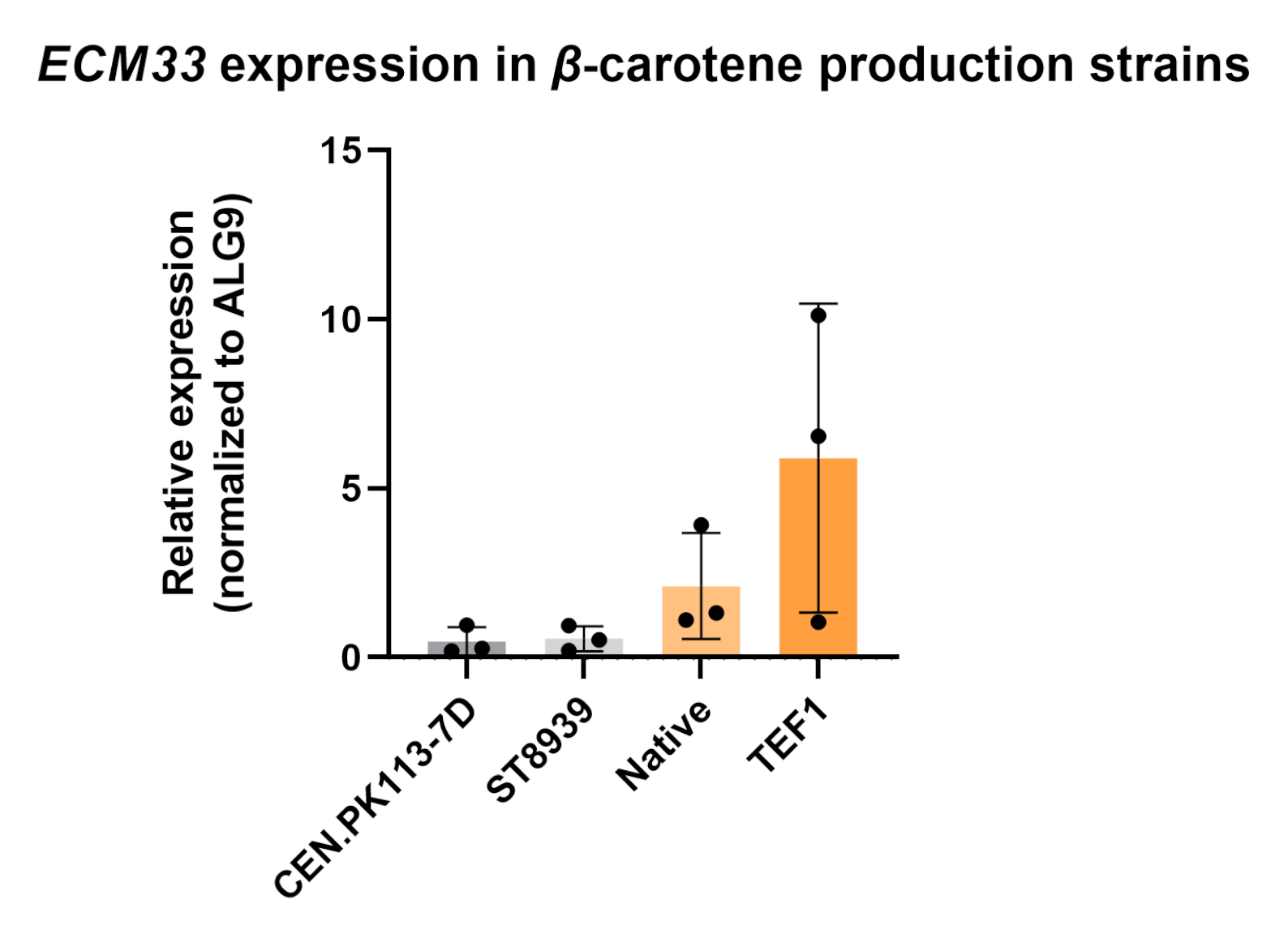

Supplement: foac037_Supplemental_files [file foac037_supplemental_files.zip › Figure_S5b.tif]
